# Broad and potent neutralizing antibodies are elicited in vaccinated individuals following Delta/BA.1 breakthrough infection

**DOI:** 10.1128/mbio.01206-23

**Published:** 2023-09-25

**Authors:** Jeffrey Seow, Zayed A. Shalim, Carl Graham, Simon Kimuda, Aswin Pillai, Thomas Lechmere, Ashwini Kurshan, Atika M. Khimji, Luke B. Snell, Gaia Nebbia, Christine Mant, Anele Waters, Julie Fox, Michael H. Malim, Katie J. Doores

**Affiliations:** 1 Department of Infectious Diseases, School of Immunology and Microbial Sciences, King’s College London, London, United Kingdom; 2 Department of Infectious Diseases, Centre for Clinical Infection and Diagnostics Research, Guy’s and St Thomas’ NHS Foundation Trust, London, United Kingdom; 3 Department of Infectious Diseases, Infectious Diseases Biobank, School of Immunology and Microbial Sciences, King’s College London, London, United Kingdom; 4 Harrison Wing, Guy's and St Thomas’ NHS Foundation Trust, London, United Kingdom; Dartmouth College, Hanover, New Hampshire, USA

**Keywords:** SARS-CoV-2, neutralizing antibodies, monoclonal antibodies, immunization, infectious disease

## Abstract

Despite the success of COVID-19 vaccines in preventing infection and/or severe disease, there has been an increase in SARS-CoV-2 infections in vaccinated individuals owing to the waning vaccine-derived immunity, and the emergence of new variants which encode escape mutations in Spike. Following breakthrough infection in vaccinated individuals, an increase in neutralization breadth has been observed in sera/plasma. However, how exposure to a heterologous Spike broadens the neutralizing response at the monoclonal antibody (mAb) level is not fully understood. Through isolation of 119 mAbs from three individuals receiving two doses of BNT162b2 vaccine before becoming Delta or Omicron/BA.1 infected, we show that serum breadth occurs due to the presence of somatically mutated mAbs with broad neutralization activity indicative of re-activation and maturation of B cells generated through previous COVID-19 vaccination. Isolated mAbs frequently show reduced neutralization of current circulating variants including BA.2.75.2, XBB, XBB.1.5, and BQ.1.1 confirming continuous selective pressure on Spike to evolve and evade neutralization. However, isolation of mAbs that display effective cross-neutralization against all variants indicates the presence of conserved epitopes on the receptor binding domain and a lesser extent the N-terminal domain. These findings have implications for the selection of Spike antigens for next-generation COVID-19 vaccines.

**IMPORTANCE:**

With the emergence of SARS-CoV-2 viral variants, there has been an increase in infections in vaccinated individuals. Here, we isolated monoclonal antibodies (mAbs) from individuals experiencing a breakthrough infection (Delta or BA.1) to determine how exposure to a heterologous Spike broadens the neutralizing antibody response at the monoclonal level. All mAbs isolated had reactivity to the Spike of the vaccine and infection variant. While many mAbs showed reduced neutralization of current circulating variants, we identified mAbs with broad and potent neutralization of BA.2.75.2, XBB, XBB.1.5, and BQ.1.1 indicating the presence of conserved epitopes on Spike. These results indicate that variant-based vaccine boosters have the potential to broaden the vaccine response.

## INTRODUCTION

Both SARS-CoV-2 infection and COVID-19 vaccines based on the SARS-CoV-2 surface glycoprotein, Spike, generate neutralizing antibodies in SARS-CoV-2 naïve individuals which can prevent infection and/or severe disease. Indeed, induction of neutralizing antibodies is a correlate of protection (1 – 4). Through isolation of monoclonal antibodies (mAbs) from SARS-CoV-2 convalescent donors or COVID-19 vaccines, we and others have identified several neutralizing epitopes on Spike (5 – 12), including epitopes on the receptor binding domain (RBD), N-terminal domain (NTD), S1D domain of S1 and on S2. mAbs against many of these epitopes have been shown to protect from SARS-CoV-2 infection in animal challenge models (13 – 15).

However, with the waning of vaccine-induced immunity (16, 17) and the emergence of SARS-CoV-2 variants of concern (VOCs) which encode mutations in Spike (18), there has been an increase in infections with VOCs in vaccinated individuals. We and others have previously shown that a breakthrough infection (BTI) with a VOC following vaccination can broaden the neutralization capacity of the polyclonal response in sera, and generate neutralizing activity against highly divergent SARS-CoV-2 viral variants carrying Spike mutations across multiple neutralizing epitopes (19 – 23). Despite the increase in infections with new VOCs, vaccines based on the ancestral SARS-CoV-2 (Wuhan-1) have remained effective at reducing severe disease and hospitalizations (24, 25). For continued control of the SARS-CoV-2 pandemic, it is important to understand how infection with SARS-CoV-2 variants in vaccinated individuals shapes the antibody response against SARS-CoV-2 Spike and the resulting susceptibility to infection with newly arising VOCs. Further understanding in this area has direct application to selecting Spike antigens to be used in future generation COVID-19 vaccines.

In the context of influenza, secondary infection with an antigenically distinct influenza strain generates antibodies that are highly cross-reactive with the primary infecting virus (termed original antigenic sin or immune imprinting) (26 – 28). This is thought to arise due to preferential induction of antibodies with higher affinity to the priming antigen than the boosting antigen. A third COVID-19 vaccine dose based on the Wuhan-1 Spike has also been shown to increase neutralization breadth against VOCs, in particular against Omicron/BA.1 (8, 19, 29, 30). However, whether a SARS-CoV-2 variant infection in vaccinated individuals leads to a *de novo* response specific for the infecting VOC or whether pre-existing memory B cells are re-activated upon VOC exposure and then undergo continued maturation that broadens reactivity is not fully understood.

Here, we isolated mAbs from three individuals who had received two doses of the BNT162b2 vaccine and then experienced a Delta or Omicron/BA.1 infection to understand how neutralization breadth increases following BTI at the mAb level. We used antigen-specific B cell sorting with an S1 probe matching the vaccine and infecting variant to isolate 119 mAbs from three individuals. We show that all isolated S1-reactive mAbs can bind and neutralize vaccine and infection strains with the majority of neutralizing mAbs targeting the RBD. Isolated mAbs showed higher levels of divergence from germline compared to mAbs isolated following double COVID-19 vaccination indicative of re-activation and continued maturation of B cell clones generated through previous vaccination. Isolated mAbs showed strong cross-neutralization of Omicron sub-lineages BA.1, BA.2, and BA.4/5 but the majority showed reduced neutralization against newer variants, including BA.2.75.2, XBB, XBB.1.5, and BQ.1.1. However, subsets of mAbs with broad cross-neutralization were identified highlighting the presence of conserved neutralizing epitopes across antigenically distant Spikes. These findings have implications for selecting Spike antigens for next-generation COVID-19 vaccines.

## RESULTS

### Wuhan-1 and VOC S1 reactive B cells present at similar levels

**Fig 1:**
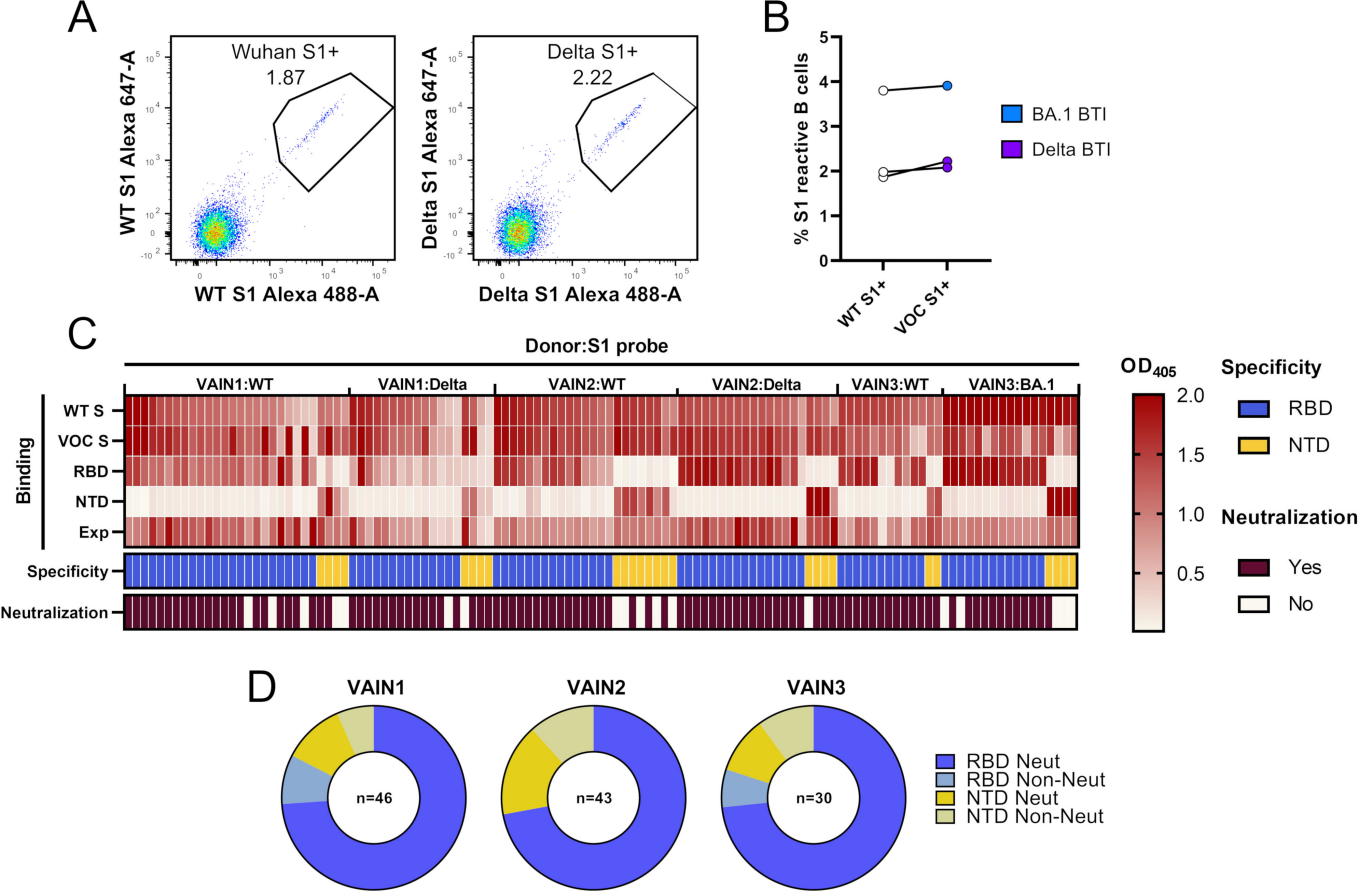
Isolation of mAbs using antigen-specific B cell sorting. (A) CD14^−^/CD3^−^/CD8^−^/CD19^+^/IgM^−^/IgD^−^/IgG^+^ and S1^+^ B cells were sorted into 96-well plates. Example: fluorescent-activated cell sorting showing percentage of CD19^+^IgG^+^ B cells binding to S1 of Wuhan-1 or S1 of Delta VOC. Full sorting gating strategy is shown in Fig. S1. (B) Percentage of CD19^+^IgG^+^ S1 Wuhan and S1 VOC reactive B cells for each donor (Delta for VAIN1 and VAIN2, BA.1 for VAIN3). Data points from the same individuals are linked. Blue: BA.1/Omicron infected and purple: Delta infected. (C) Heatmap showing IgG expression level and binding to SARS-CoV-2 Spike [WT and VOC (Delta for VAIN1 and VAIN2, BA.1 for VAIN3)], and to Spike domains RBD and NTD. The figure reports optical density (OD) values from a single experiment (range 0–2.0) for undiluted supernatant from small-scale transfection of 119 cloned mAbs. Antigen binding was considered positive when OD at 405 nM was >0.2 after subtraction of the background. SARS-CoV-2 Spike domain specificity (RBD or NTD) for each antibody is indicated. Neutralization activity was measured against wild-type (Wuhan) pseudotyped virus using concentrated supernatant and neutralization status is indicated. Antigen probe used to select specific B cells is indicated (i.e., WT S1, Delta S1, or BA.1 S1). (D) Distribution of mAbs targeting RBD and NTD for each donor, as well as their neutralization capability. mAbs are classified as shown in the key (related to Fig. S1 and S2; Table S1).

To gain insight into the neutralizing activity within polyclonal sera from BTI individuals, we used antigen-specific B cell sorting to isolate S1-reactive IgG^+^ B cells (see Fig. S1 for full sorting strategy). Participants VAIN1 and VAIN2 were infected during the UK Delta wave (11 August 2021 and 23 August 2021, respectively), and participant VAIN3 was infected during the UK BA.1 wave (18 December 2021) (31). Viral sequencing was not performed on these samples. All three donors had no reported history of SARS-CoV-2 infection prior to the breakthrough infection and had received two doses of the BNT162b2 vaccine with an extended interval (19) prior to infection. Blood samples were collected 15, 87, and 26 days post-SARS-CoV-2 infection, for VAIN1, VAIN2, and VAIN3, respectively (see Table S1 for full donor information). Cross-neutralizing activity against wild type (WT), Delta, Beta, BA.1, BA.2, and BA.4 was observed in sera collected at these time points (Fig. S2). An S1 probe was used for B cell sorting as the majority of neutralizing antibodies target the RBD and NTD (5, 12). To allow for the identification of variant-specific mAb responses, we performed two sorts from each donor using different antigen baits, one sort using the Wuhan-1 S1 (matched vaccine strain and referred to as WT) and one sort using the VOC S1 (Delta S1 for VAIN1 and VAIN2, and BA.1 S1 for VAIN3) (Fig. 1A). Similar levels of WT and VOC reactive IgG^+^ B cells were observed for all three donors (Fig. 1B).

mAb heavy and light chain genes were rescued using reverse transcription and nested PCR using gene-specific primers (32, 33). The variable regions were then cloned into IgG1 expression vectors using Gibson assembly and directly transfected in the HEK293T/17 cells (5, 6). Crude supernatants were tested by enzyme-linked immunosorbent assay (ELISA) and the heavy and light chain genes of Spike reactive IgGs were sequenced. In total, 46, 43, and 30 spike-reactive mAbs were isolated from VAIN1, VAIN2, and VAIN3, respectively (Fig. 1C).

### Delta and BA.1 BTI generates neutralizing mAbs against RBD and NTD

ELISA with the crude supernatants was used to determine the VOC specificity and the specific domains targeted by each mAb. Despite different antigen baits being used for B cell selection, all mAbs isolated showed reactivity to both the WT and VOC Spikes, consistent with reactivation of B cells generated from prior vaccination (Fig. 1C). Similar to previous observations (5, 6), 72.1%–83.3% of mAbs were RBD specific (Fig. 1D) with the remaining mAbs specific for NTD.

Neutralization activity of concentrated supernatant was determined using HIV-1 viral particles, pseudotyped with SARS-CoV-2 Wuhan-1 (WT) Spike (34). As previously observed, the majority (93.5%) of RBD-specific mAbs had neutralizing activity (Fig. 1D) whereas only 53.8% of NTD mAbs showed neutralizing activity against WT pseudotyped virus.

### Mutation and germline gene usage

**Fig 2:**
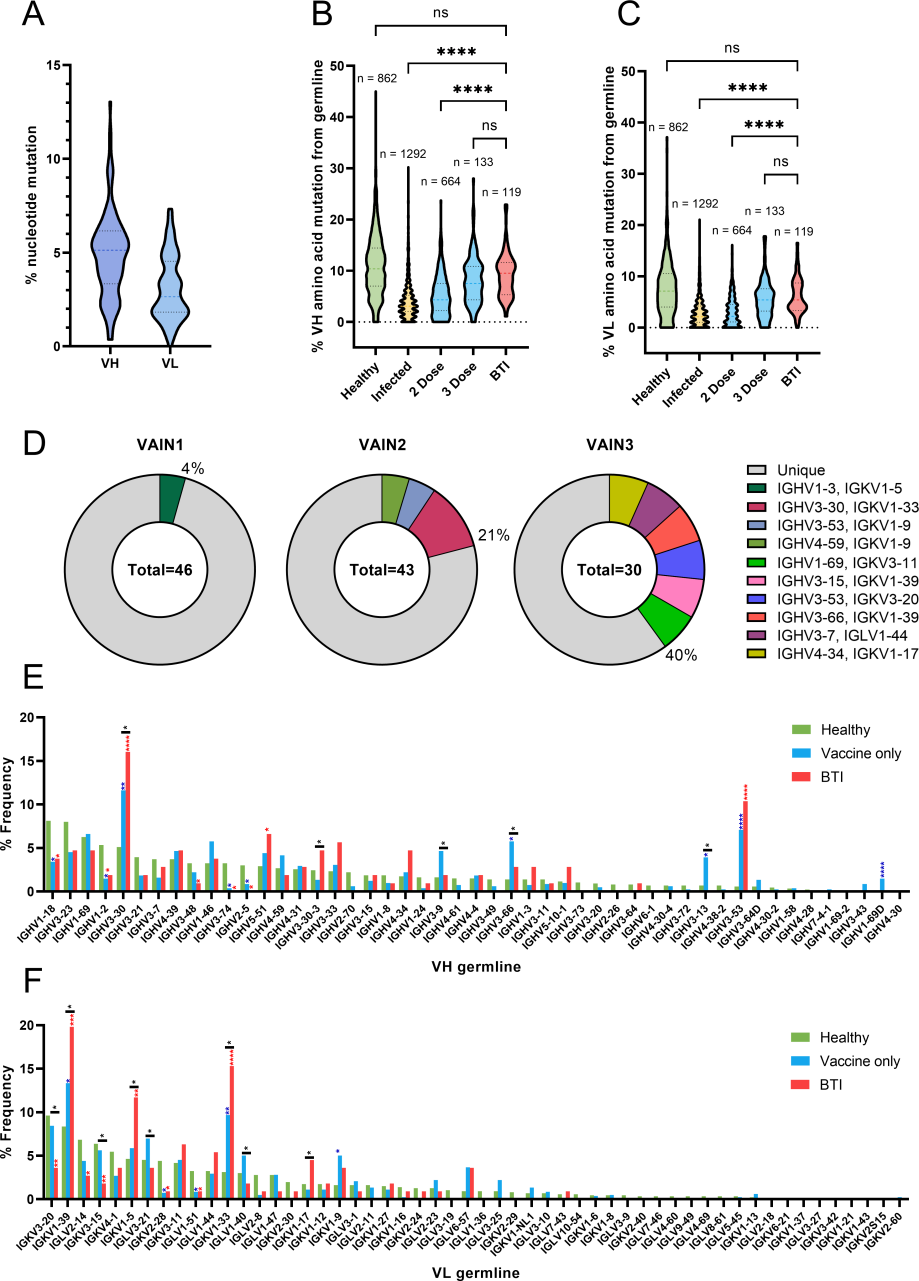
BTI mAbs show higher somatic hypermutation than mAbs isolated following two vaccine doses. (A) Truncated violin plot showing the percentage of nucleotide mutation compared with germline for the V_H_ and V_L_ genes of Spike-reactive mAbs isolated from VAIN1, VAIN2, and VAIN3. Truncated violin plot comparing the percentage of amino acid mutation compared with germline for (B) V_H_ and (C) V_L_ between Spike-reactive mAbs isolated following infection, two doses of vaccine, three doses of vaccine, or following BTI and IgG B cell receptors (BCRs) from SARS-CoV-2-naive individuals (36). D’Agostino and Pearson tests were performed to determine normality. Based on the result, a Kruskal-Wallis test with Dunn’s multiple comparison post hoc test was performed. *P < 0.0332, **P < 0.0021, ***P < 0.0002, and ****P < 0.0001. (D) Pie chart showing the distribution of heavy chain sequences for donors VAIN1, VAIN2, and VAIN3. The number inside the circle represents the number of heavy chains analyzed. The Pie slice size is proportional to the number of clonally related sequences and is color coded based on clonal expansions described in Table S2. The percentage (%) on the outside of the Pie slice represents the overall % of sequences related to a clonal expansion. Graph showing the relative abundance of (E) *V*_*H*_ and (F) *V*_*L*_ gene usage for Spike-reactive mAbs isolated following infection (*n* = 1,292), vaccination (*n* = 817, including two and three vaccine doses) or following BTI (*n* = 106), and IgG BCRs from SARS-CoV-2-naive individuals (*n* = 1,292) (36). Statistical significance was determined by binomial test. *P ≤ 0.05, **P ≤ 0.01, ***P ≤ 0.001, and ****P ≤ 0.0001. Blue stars represent vaccine vs healthy, red stars represent BTI vs healthy, and black stars represent BTI vs vaccine (related to Fig. S3; Table S2).

The level of somatic hypermutation and germline gene usage was determined using the IMGT database (35). The mean divergence from germline at the nucleotide level for the variable heavy (*V*_*H*_) and light (*V*_*L*_) regions was 5.0% and 3.9%, respectively (Fig. 2A). Comparison of mutation levels between the three donors showed that VAIN3 (BA.1 infected) had higher mutation than VAIN1 and VAIN2 in the *V*_*H*_ and *V*_*L*_ regions (Fig. S3A). mAbs selected using the BA.1 S1 probe were more mutated than Delta or WT S1 selected B cells (Fig. S3B). However, this might be a donor-specific observation as there was no difference in the level of mutation in *V*_*H*_ between WT S1 and VOC S1 selected B cells from each donor (Fig. S3C).

The degree of divergence from germline was also compared to a database of previously published SARS-CoV-2 Spike-specific mAbs isolated from convalescent donors (*n* = 1,292) and individuals who had received two doses (*n* = 664) or three doses (*n* = 133) of a COVID-19 vaccine [vaccines included mRNA (*n* = 489), viral vectored (AZD1222) (*n* = 89), and AD5-nCoV (*n* = 6)] and inactivated vaccines [SinoVac (*n* = 118) as well as mixed prime/boost regimens (*n* = 72)] (37). The SARS-CoV-2 mAb database is a public database including published and patented mAbs from many research groups (https://opig.stats.ox.ac.uk/webapps/covabdab/). Divergence was also compared with paired heavy and light chains of IgG B cell receptors from CD19^+^ B cells of healthy individuals (*n* = 862) (36) (Fig. 2B and C). Since the SARS-CoV-2 mAb database only included amino acid sequences for some mAbs, divergence from germline was determined at the amino acid level, which correlated well with nucleotide divergence (Fig. S3D). BTI S1-reactive mAbs had a statistically higher amino acid mutation level (*V*_*H*_ 9.2% and *V*_*L*_ 6.2%) compared to mAbs isolated following infection only (*V*_*H*_ 4.2% and *V*_*L*_ 3.0%) and following two vaccine doses (*V*_*H*_ 5.3% and *V*_*L*_ 3.2%). However, there was no statistical difference in mutations levels between BTI mAbs and mAbs isolated following three vaccine doses (*V*_*H*_ 8.2% and *V*_*L*_ 5.6%) indicating an additional exposure to SARS-CoV-2 Spike in the form of infection or vaccination leads to increased somatic hypermutation. Non-Spike-specific B cells were more highly mutated than BTI mAbs (*V*_*H*_ 10.9% and *V*_*L*_ 7.5%). Comparison of the CDRH3 length distribution of BTI mAbs with representative naive repertoires (38) showed an enrichment in CDRH3 of lengths 20 amino acids (Fig. S3E) which is predominantly driven by a clonal expansion of a VH3-30 germline family from VAIN2 (Table S2).

Sequence analysis identified clonally related sequences within all three donors, representing 4%, 21%, and 40% of all B cells from VAIN1, VAIN2, and VAIN3, respectively (Fig. 2D; Table S2). WT and VOC S1 probes pulled out clonally related mAbs independently from VAIN2 and VAIN3 donors (Table S2) demonstrating their cross-reactive nature. Germline usage of BTI mAbs was also compared with non-Spike reactive mAbs (*n* = 862) and vaccine-derived mAbs within the SARS-CoV-2 mAb database (*n* = 817) (Fig. 2E and F). As previously observed, there was an enrichment in VH3-53 and VH3-30/VH3-30-3 germline usage for BTI mAbs (Fig. 2E) (5, 39 – 42). mAbs utilizing these VH3-53 typically target the ACE2-binding site on RBD (5, 39 – 42). VH3-30/VH3-30-3 encoded 20 RBD-specific BTI mAbs with neutralizing activity (Table S3). Enrichment in VH5-51 was seen for BTI mAbs compared to non-Spike mAbs from naïve donors but this was not seen for vaccine-derived mAbs. When comparing between vaccine-derived mAbs and BTI mAbs, enrichments in VH3-9, VH3-66, and VH3-13 were seen for vaccine-derived mAbs but not for BTI mAbs. When considering the light chain, enrichment in gene usage was seen for VK1-39 (22/119), VK1-5 (12/119), and VK1-33 (18/119), and enrichment of these germlines was greater than observed for vaccine-derived mAbs. Overall, there continues to be a diverse germline usage for SARS-CoV-2-specific mAbs present following infection in vaccinated individuals.

### mAbs isolated following BTI have broad neutralization against Omicron sub-lineages

**Fig 3:**
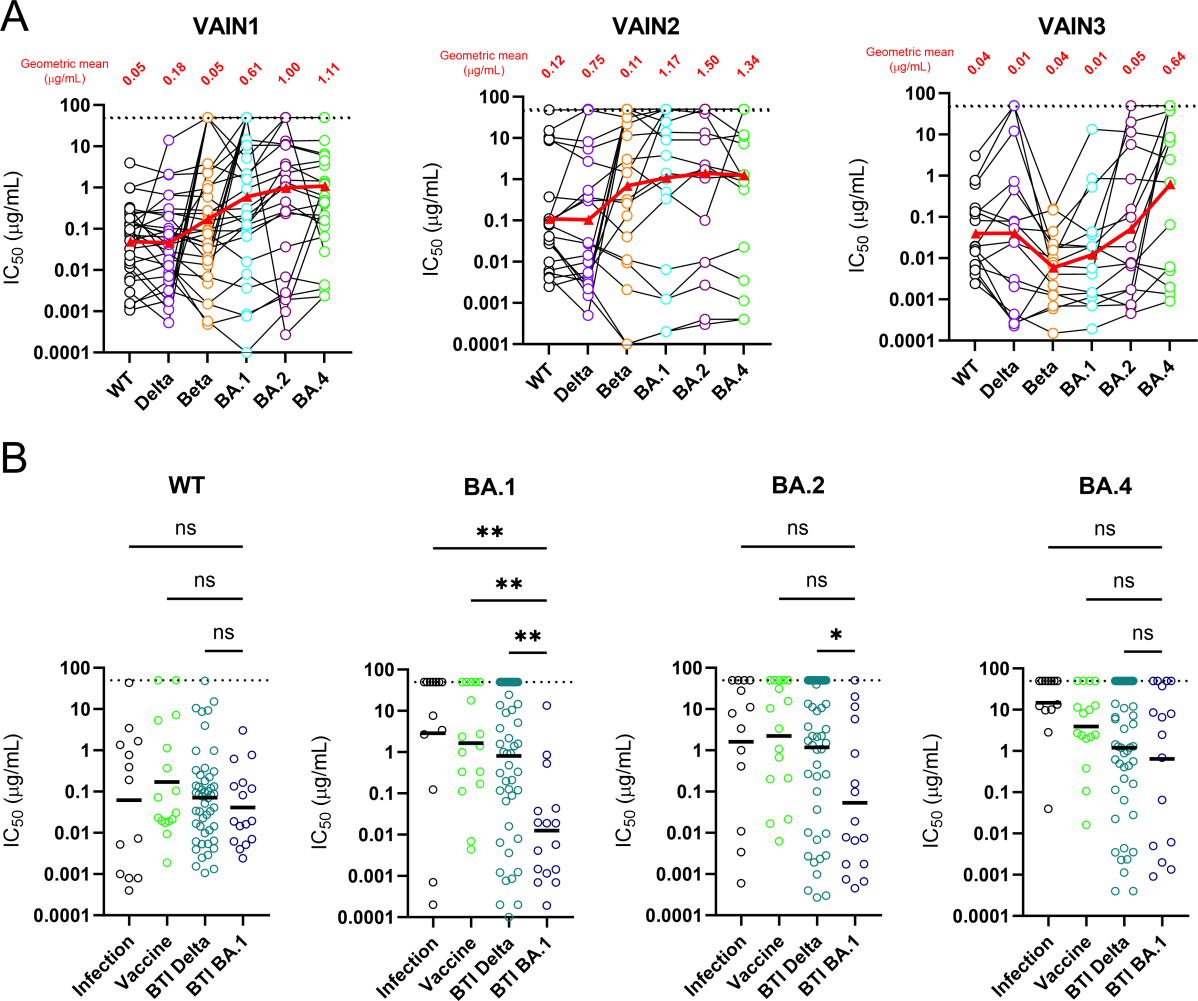
Neutralization breadth and potency against Omicron sub-lineages. (A) Neutralization breadth and potency of BTI mAbs against Wuhan-1, Delta, Beta, BA.1, BA.2, and BA.4 from VAIN1, VAIN2, and VAIN3. Data for each mAb are linked. Red triangle and linking line show the geometric mean IC_50_ against each variant. Dotted line represents the highest concentration of antibody tested. (B) Comparison of neutralization breadth and potency of BTI mAbs with mAbs isolated from convalescent donors (infection) (5) and an AZD1222 vaccinated donor (6) against Omicron sub-lineages (BA.1, BA.2, and BA.4). Horizontal line represents the geometric mean IC_50_ against each mAb origin. D’Agostino and Pearson tests were performed to determine normality. Based on the result, a Kruskal-Wallis test with Dunn’s multiple comparison post hoc test was performed. *P < 0.0332, **P < 0.0021, ***P < 0.0002, and ****P < 0.0001 (related to Table S3).

Sixty-seven RBD- and NTD-specific neutralizing antibodies from the three donors utilizing a range of germlines were selected for large-scale expression and purification to allow further characterization of neutralization breadth, potency, and epitope specificity. Neutralization of purified mAbs was measured against a panel of viral particles pseudotyped with different SARS-CoV-2 variant Spikes, including WT, Delta, Beta, BA.1, BA.2, and BA.4/5 (Table S4). mAbs with potent activity against all six viruses tested were identified in all three donors (Fig. 3A). The neutralization potency of mAbs against WT and BTI variants correlated well for both Delta BTI and BA.1 BTI mAbs (Fig. S4A). When considering the geometric mean IC_50_ (half-maximal inhibitory concentration) for mAbs isolated from VAIN1 and VAIN2 (Delta infection) and VAIN3 (BA.1 infection), all three donors had similar mean IC_50_ values against WT and Delta. However, a different pattern of potencies was observed for other variants (Fig. 3A). Whereas WT and Delta were most potently neutralized by mAbs from the Delta-infected donors, the BA.1 and Beta were most potently neutralized by mAbs from the BA.1-infected donor. BA.1 and Beta share common mutations in RBD (K417N, E484K, N501Y) which could explain the high level of cross-reactivity of mAbs from VAIN3 with the Beta variant.

The neutralization breadth of mAbs isolated following BTI was compared to that of mAbs which we previously isolated from convalescent donors early in the pandemic (March to May 2020) (5) and mAbs isolated following two doses of COVID-19 vaccine (AZD1222) administered with a 12-week interval between doses (6). Analysis was focused on Omicron sub-lineages BA.1, BA.2, and BA.4/5 (Fig. 3B). The geometric mean IC_50_s against WT pseudotyped virus were most similar among the three mAb groups, whereas neutralization of the Omicron sub-lineages showed larger differences. The least potent neutralization was observed by infection and vaccine mAbs against BA.1, BA.2, and BA.4/5. mAbs isolated following Delta BTI had similar geometric mean titre (GMT) against BA.1, BA.2, and BA.4/5, whereas mAbs isolated following BA.1 BTI were more potent at neutralizing BA.1 compared to BA.2 and BA.4/5. The greater neutralization breadth of BTI mAbs is consistent with higher divergence from germline sequence (6, 23, 43 – 45). Neutralization potency (IC_50_) of BTI mAbs from VAIN1 and VAIN2 did not correlate with the level of somatic hypermutation, whereas neutralization potency (IC_50_) against WT, Beta, Delta, and BA.2 correlated with higher levels of mutation for BTI mAbs from VAIN3 (Fig. S4B). Interestingly, some of the mAbs isolated from the convalescent and vaccinated donors showed potent cross-neutralization against all Omicron sub-lineages (46) despite having only experienced the WT Spike. Overall, mAbs with potent cross-neutralization were identified against antigenically distinct Omicron sub-lineages.

### RBD-specific mAbs form five competition groups

**Fig 4:**
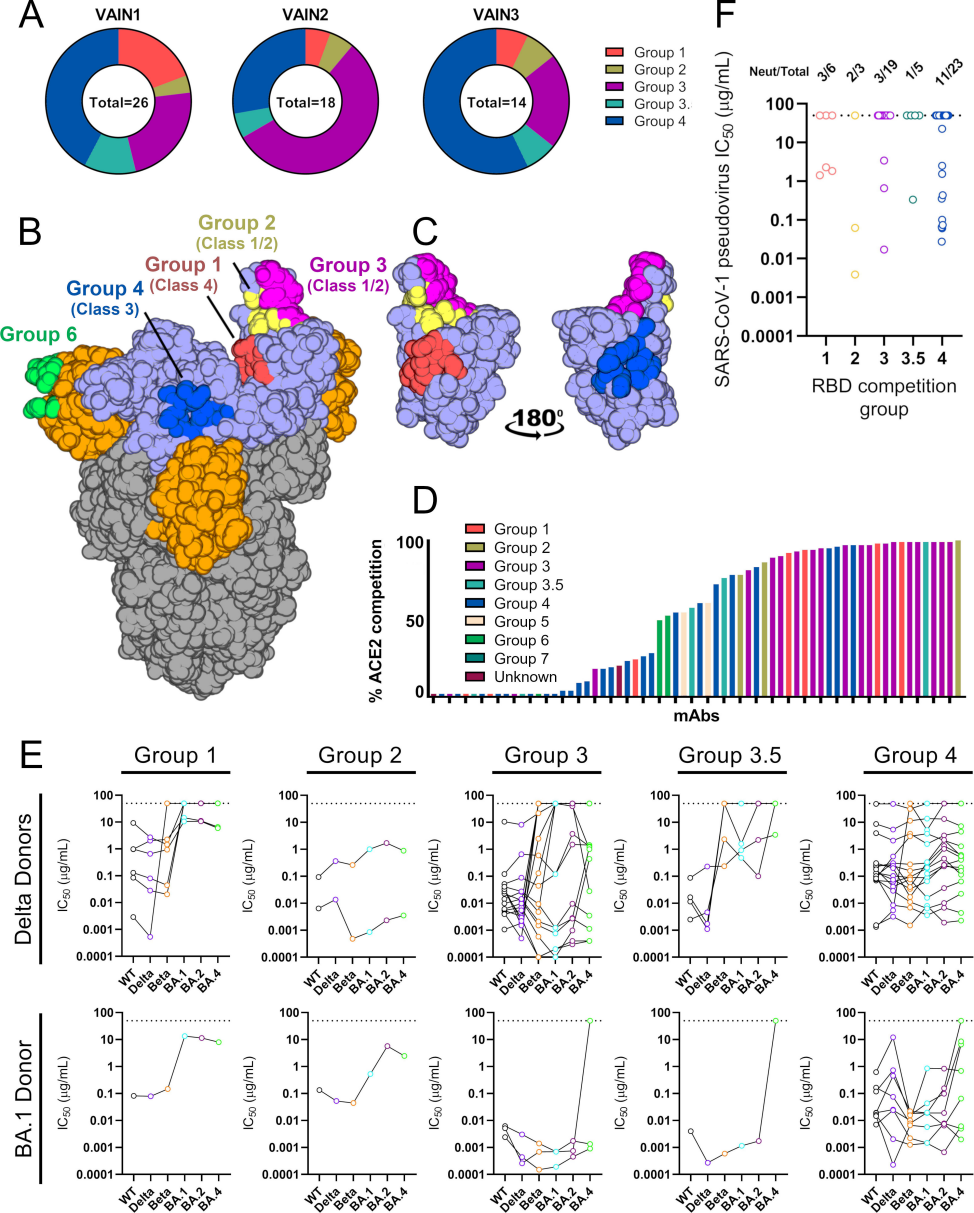
RBD mAb characterization. (A) Pie chart showing distribution of RBD-specific mAbs between competition groups for VAIN1, VAIN2, and VAIN3. (B) Surface representation of SARS-CoV-2 WT spike (pdb:6XM0) showing epitopes of previously characterized competition groups as colored surfaces (6). RBD competition groups are also shown as RBD classes as defined by Barnes et al. (40). RBD and NTD are indicated by light blue and orange, respectively. (C) Surface representation of RBD domain in the up conformation showing location and proximity of Group 1 (red), Group 2 (yellow), Group 3 (magenta), and Group 4 (blue). Structures were generated in Pymol. (D) Ability of RBD-specific neutralizing antibodies to inhibit the interaction between cell surface ACE2 and soluble SARS-CoV-2 Spike. mAbs (at 600 nM) were pre-incubated with fluorescently labeled Spike before addition to HeLa-ACE2 cells. The percentage reduction in mean fluorescence intensity is reported. Experiments were performed in duplicate. Bars are color coded based on their competition group. (E) Neutralization breadth and potency of RBD-specific mAbs within the different RBD competition groups. mAbs are separated by the infecting VOC. Data for each mAb are linked. Dotted line represents the highest concentration of antibody tested. (F) Neutralization potency of RBD-specific mAbs against SARS-CoV-1. Data are presented by RBD competition group (related to Fig. S4 through S6).

To understand more about the epitopes targeted on RBD, we performed Spike competition ELISAs between neutralizing antibodies with known RBD specificity that had been isolated from convalescent or vaccinated donors (5, 6) (Fig. 4A through C; Fig. S5A). Furthermore, to gain insight into mechanisms of neutralization, the ability of mAbs to inhibit the binding of soluble Spike to HeLa-ACE2 cells was measured by flow cytometry (5) (Fig. 4D). mAbs with high inhibition levels directly block ACE2 binding through binding to the receptor binding motif (RBM) (5, 6). The competition ELISA revealed that the RBD-specific mAbs formed five competition groups (Fig. S5A), four of which had been observed previously (5, 6, 40) and matched with RBD classes reported by Barnes et al. (Fig. 4B) (40). The distribution of RBD-specific mAbs between competition groups differed between the three donors, with VAIN1 and VAIN3 having the highest frequency of competition Group 4 and VAIN2 having the highest frequency of Group 3 (Fig. 4A). Our previous studies isolating mAbs following infection (5) or vaccination (6) had shown a dominance of Group 3 and Group 4 RBD-specific mAbs, respectively. Interpretation of the biological significance of the differences in epitope immunodominance is limited due to the small number of mAbs studied.

The majority of Group 1 mAbs [RBD Class 4 (40)], which bind an epitope distal to RBM (Fig. 4B and C), showed neutralization activity against WT, Delta, and Beta VOCs, but had greatly reduced or limited neutralization activity against the Omicron sub-lineages (Fig. 4E). This was true for mAbs isolated following both Delta and BA.1 BTI. Group 2 mAbs (RBD Class 1/2 (40)), characterized by their ability to compete with both Group 1 and Group 3 mAbs, showed strong ACE2 competition (Fig. 4D) as well as cross-neutralization of VOCs.

Group 3 mAbs [RBD Class 1/2 (40)] were enriched with VH3-53/3-66 germline usage (11/18) (Table S3) which have been shown to bind the ACE2 RBM on RBD (40 – 42). Indeed, the majority of Group 3 mAbs showed >90% inhibition of ACE2 binding (Fig. 4D). Interestingly, several mAbs that competed strongly with Group 3 mAbs showed very little inhibition of ACE2 binding suggesting a wide Spike footprint for this competition group and differing angles of approach. VH3-53/3-66 using mAbs showed broad and potent neutralization of Omicron sub-lineages reaching IC_50_ < 0.001 µg/mL (Fig. 4E). However, Group 3 VH3-30 using mAbs isolated following Delta BTI had limited neutralization breadth and only neutralized WT and Delta VOCs (Table S3). Loss of neutralization against Beta and Omicron sub-lineages is likely due to mutations within the RBM site.

mAbs within Group 4 [RBD Class 3 (40)] competed with mAbs known to bind distal to the RBM and able to bind the RBD in its closed conformation (Fig. 4B). These mAbs showed broad cross-neutralization across VOCs (Fig. 4E) but the overall neutralization potency was reduced against WT and Delta compared to the Group 3 mAbs with IC_50_ in the 0.001–8.65 µg/mL (geometric mean 0.11 µg/mL) and 0.0001–12.0 µg/mL (geometric mean 0.11 µg/mL) range for WT and Delta, respectively (Fig. S6). There was an enrichment in VH5-51 germline gene usage (6/24) (Table S3). A range of ACE2 inhibitions were observed, indicating the large epitope footprint of this competition group (Fig. 4D). An additional competition group (named Group 3.5) was identified compared to our previous studies (5, 6). Group 3.5 mAbs competed with both Group 3 and Group 4 mAbs (Fig. 4C) and, while potently neutralizing WT and Delta, showed limited neutralization of Omicron sub-lineages, in particular BA.4 (Fig. 4E).

To determine whether the epitopes of the RBD-specific mAbs isolated following BTI are conserved on other betacoronaviruses, we next measured neutralization activity against SARS-CoV-1 pseudotyped virus (Fig. 4F). Cross-neutralization of SARS-CoV-1 was observed for mAbs belonging to all five RBD competition groups, with a particular abundance within competition Group 4 (11/23). Group 2 mAb VAIN3O_12, isolated following BA.1 BTI, was most potent, neutralizing SARS-CoV-1 with an IC_50_ of 0.0039 µg/mL.

Overall, RBD mAbs competed with previously isolated RBD-specific mAbs suggesting new RBD epitopes are not being targeted. However, the increased cross-competition between competition groups suggests a larger collective RBD footprint for neutralizing antibodies. Further structural characterization is required to understand how VOC BTI influences the specificity of RBD mAbs at the molecular level.

### Binding of NTD mAbs to VOCs does not correlate with neutralization activity

**Fig 5:**
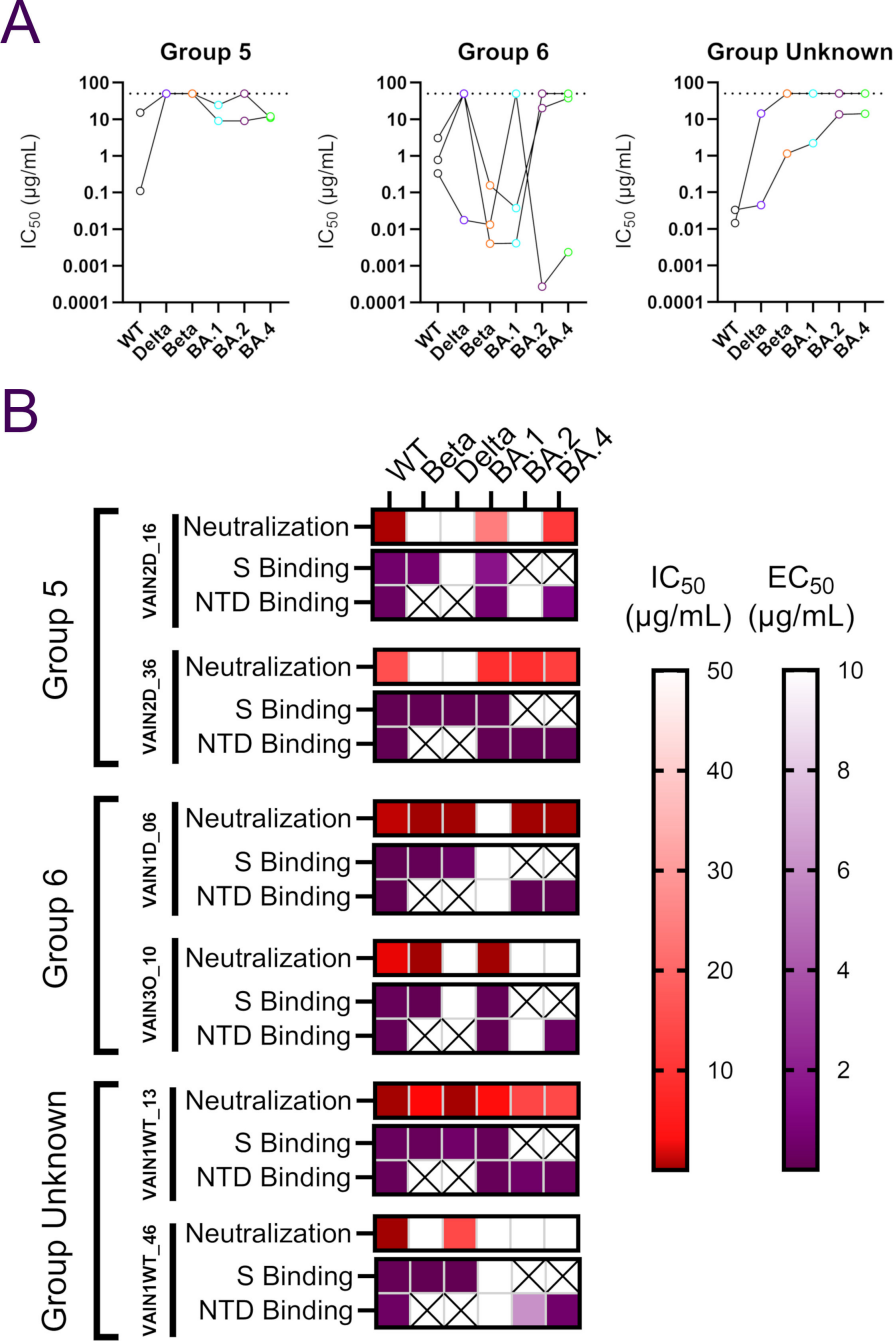
NTD mAb characterization. (A) Neutralization breadth and potency of NTD-specific mAbs within the different NTD competition groups. Data for each mAb are linked. Dotted line represents the highest concentration of antibody tested. (B) Comparison between neutralization activity (IC_50_) and binding to Spike or NTD (EC_50_) by ELISA for NTD-specific mAbs. IC_50_ and EC_50_ (half-maximal effective concentrations of binding) values are shown as a heat map for each NTD-specific mAb with the level of binding shown in the key. A cross indicates that the Spike or NTD antigen for that variant was not available to test (related to Fig. S5).

Competition for Spike binding between NTD-specific neutralizing antibodies with known specificity was used to determine the epitopes targeted by the seven NTD-specific neutralizing antibodies isolated (5). We have previously identified three NTD-specific mAb competition groups (5, 6) and structural characterization of mAb P008_56 from Group 6 revealed binding to NTD adjacent to the ß-sandwich fold (47). The NTD-specific mAbs isolated following BTI formed three competition groups (Fig. S5B). Groups 5 and 6 were identified previously, but an additional group that did not compete with previously isolated NTD mAbs was also identified (designated NTD unknown). Group 5 mAbs VAIN2D_36 and VAIN2D_16 had poor cross-neutralization of VOCs (Fig. 5A) and despite being isolated from a Delta-infected donor, they were unable to neutralize Delta. Both Group 5 mAbs utilized the VH4-34 germline but were not clonally related. While Group 6 mAbs showed greater cross-neutralization compared to Group 5 mAbs, none were able to neutralize all six VOCs (Fig. 5A) but they were able to neutralize the variant the donors were infected with. The most broad and potent Group 6 mAb was VAIN1D_06 which neutralized all VOCs, except BA.1, with IC_50_ < 0.55 µg/mL. mAb VAIN1WT_13 from the non-competing group (NTD unknown) neutralized all six variants with IC_50_ between 0.033 µg/mL and 13.9 µg/mL with the lowest neutralization potency against Omicron sub-lineages.

To determine whether NTD mAbs lacking neutralization ability against a VOC was due to an inability to bind the NTD, ELISAs were performed using recombinant Spike (WT, Delta, Beta, and BA.1) and recombinant NTD (WT, BA.1, BA.2, and BA.4) antigens (Fig. 5B). While binding and neutralization were consistent for VAIN1D_06 and VAIN1WT_13, binding did not always lead to neutralization for other NTD-specific mAbs. For example, Group 5 mAbs VAIN2D_16 and VAIN2D_36 bound well to recombinant Beta Spike but did not neutralize Beta pseudovirus. Furthermore, mAb VAIN1WT_46 bound to BA.2 and BA.4 NTD but did not neutralize the corresponding viral particles. This disconnect between NTD binding and neutralization was also observed by Wang et al. (48). Mechanisms of NTD-specific mAb neutralization are not fully understood. However, the high mutation level in this region suggests NTD is under strong selective pressure from the host’s humoral immune response. McCallum et al. demonstrate that some mAbs targeting the NTD supersite prevent SARS-CoV-2 Spike-mediated cell-cell fusion (13), while Cerutti et al. showed that NTD mAbs use a restricted angle of approach to facilitate neutralization (49). It is possible that the mutations, and/or insertions and deletions, within NTD encoded by different VOCs may alter the angle of approach which in turn reduces neutralization capability. Whether cross-binding but non-neutralizing NTD-specific mAbs can facilitate effector functions through their Fc receptors needs to be investigated further (50, 51).

### XBB, XBB.1.5, BA.2.75.2, and BQ.1.1 show greater antigenic divergence

**Fig 6:**
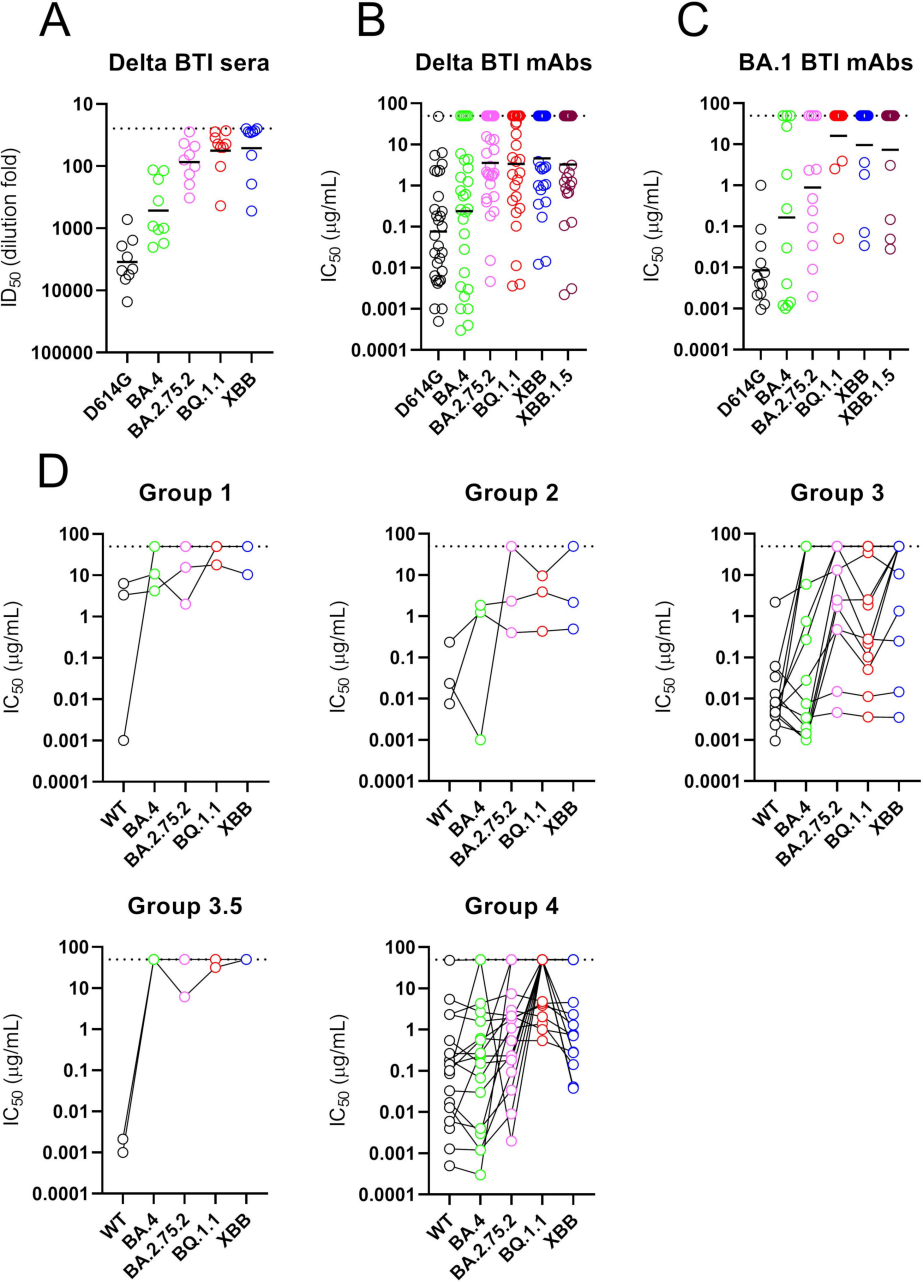
mAb neutralization against more recent VOCs including BA.2.75.2, XBB/XBB.1.5, and BQ.1.1. Neutralization by (A) plasma from individuals vaccinated with two doses of BNT162b2 and were subsequently Delta infected (ID_50_), (B) mAbs from Delta BTI donors (IC_50_), and (C) mAbs from BA.1 BTI donor (IC_50_). Sera were collected 15–35 days post-infection. Additional plasma/sera samples from double vaccinated and BA.1-infected individuals were not available. Horizontal line represents geometric mean IC_50_ (for mAbs) or geometric mean titers (sera). (D) Neutralization breadth and potency broken down by RBD competition group. Data for each mAb are linked. Dotted line represents the highest concentration of mAb or lowest dilution of sera tested (related to Fig. S7).

SARS-CoV-2 Spike continues to acquire mutations. Since the Omicron waves (including BA.1, BA.2, BA.4, and BA.5) new VOCs that have emerged include BA.2.75.2 (evolved from BA.2), XBB and XBB.1.5 (a recombinant of two BA.2 lineages, BA.2.75 and BJ.1), and BQ.1.1 (evolved from BA.5) (Table S4). Despite these variants being on divergent evolutionary courses, they share convergent mutations in RBD. Additional mutations in RBD compared to BA.1 include R326T and N460K in BA.2.75.2, XBB/XBB.1.5 and BQ.1.1, G446S and F486S in BA.2.75.2 and XBB/XBB.1.5, and K444T in BQ.1.1. A panel of mAbs was selected, based on their neutralization activity against Omicron sub-lineages, to gain insight into whether BTI following two vaccine doses could elicit antibodies capable of neutralizing these new variants (Fig. 6B and C). Neutralization potencies were compared to the neutralization activity in sera from the three donors (Fig. S7) as well as with a larger group of double vaccinated individuals experiencing a Delta BTI (Fig. 6A).

Whereas the sera from donors VAIN1, VAIN2, and VAIN3 had shown broad cross-neutralization of Omicron sub-lineages (Fig. S2), there was a reduction in neutralization of BA.2.75.2, XBB, XBB.1.5, and BQ.1.1 (Fig. S7). This pattern of neutralization was observed in the larger panel of sera tested (Fig. 6A) as well as by the isolated mAbs (Fig. 6B and C). While many of the BTI mAbs had retained some level of neutralization activity against the Omicron sub-lineages, many of the mAbs tested lost neutralization activity against all four VOCs. However, RBD-specific mAbs with cross-neutralizing activity against all variants were still identified and importantly these belonged to multiple RBD competition groups (including Group 2, Group 3, and Group 4) (Fig. 6D) indicating that the additional Spike mutations did not lead to complete disruption of all RBD neutralizing epitopes. Group 3 mAbs, VAIN2D_12 and VAIN2D_17, were most potent, neutralizing all VOCs with IC_50_ < 0.01 µg/mL (Table S3). Other cross-neutralizing RBD-specific antibodies were less potent, only reaching an IC_50_ between 0.1 µg/mL and 10 µg/mL. Whether mAbs with cross-neutralizing activity could undergo further mutation to enhance neutralization potency would be of interest for optimization of mAbs for therapy against diverse VOCs.

Overall, despite broad neutralization of BA.1, BA.2, and BA.4, the convergent RBD mutations in BA.2.75.2, XBB/XBB.1.5, and BQ.1.1 lead to extensive immune evasion to mAbs generated following Delta and BA.1 BTI. Several potent cross-neutralizing mAbs were identified and additional structural studies would provide important insights into how these mAbs tolerate these additional RBD mutations.

## DISCUSSION

Studies conducted by us and others using convalescent sera or plasma have shown that a Delta or BA.1 infection following COVID-19 vaccination can broaden the neutralization activity against Omicron sub-lineages (19, 20, 22, 52, 53). Through isolation of mAbs from BNT162b2 double vaccinated individuals that were subsequently Delta or BA.1 infected, we showed that this increase in neutralization breadth is due to the presence of mAbs with potent cross-neutralizing activity. Despite using antigen baits specific for the vaccine and the infecting variant, we observed similar levels of WT- and VOC-specific B cells and did not identify mAbs that were specific for the infecting variant. Combined with the observation that BTI mAbs had a higher level of somatic hypermutation compared to double-vaccine and infection-only elicited mAbs, we infer that Delta or BA.1 infection in vaccinated individuals predominantly resulted in re-activation and maturation of B cells generated through previous COVID-19 vaccination. This is consistent with findings from several other recent studies on breakthrough infection (23, 54, 55). Specifically, Ellebedy and co-workers showed, using longitudinal samples, that the B cell response following breakthrough infection predominantly resulted in boosting existing memory B cell responses (55). However, they were also able to isolate rare variant-specific B cells that were also identified as arising from a *de novo* response specific to the VOC Spike. We and others have shown that sera from individuals whose first SARS-CoV-2 exposure was a Delta infection showed strong homologous neutralization but also low levels of VOC cross-neutralization (20, 56, 57). Therefore, it is possible that cross-neutralizing mAbs also arose from the activation of new B cells expressing cross-reactive B cell receptors.

With the indication of reactivation of existing B cells, it might be expected that prior COVID-19 vaccinations based on Wuhan-1 might limit neutralization breadth of mAbs in a manner similar to that observed following influenza re-exposure (26 – 28). However, the continued maturation upon re-activation of B cells appears to lead to mAbs with increased neutralization breadth. This observation is supported by research showing that wider SARS-CoV-2 neutralization breadth is associated with increased antibody somatic hypermutation (6, 23, 43 – 45). The three donors studied here had received two vaccine doses prior to infection with an antigenically distinct Spike (either Delta or BA.1). A third vaccine dose based on the Wuhan-1 strain has also been shown to increase neutralization breadth within polyclonal sera/plasma (19, 58, 59) and mAbs isolated from such individuals also show continued maturation and increased neutralization breadth and potency against VOCs, in particular BA.1 (29, 30, 60). Taken together, these findings show that a third antigenic stimulation, independent of the Spike variant, can increase neutralization breadth. However, studies examining the impact of a fourth antigenic stimulation show a more modest increase in neutralization breadth and potency of isolated mAbs (10).

This study has implications for variant-based vaccine boosters. COVID-19 vaccine boosters are important for maintaining circulating levels of antibodies as well as providing broadened protection against newly emerging variants. While the goal of variant-based vaccine boosters is to match circulating strains dominant in the human population, this study suggests that exposure to an antigenically distinct Spike (either Delta or BA.1) can provide broad protection through generating mAbs with cross-neutralizing activity instead of a variant-specific mAb response. Indeed, bivalent vaccine boosters based upon the BA.1 or BA.4/5 Spike antigens are now being used and are effective at generating broad neutralization against Omicron sub-lineages similar to monovalent boosters (61, 62) and at preventing severe disease following BA.4.6, BA.5, BQ.1, and BQ.1.1 infections (63).

Many BTI mAbs could neutralize variants of concern which have diverged independently from the ancestral Wuhan-1 strain and previous studies using a variety of immune sera have highlighted their antigenic distance (56, 64). This cross-neutralization highlights that despite large variation in Spike, several conserved neutralizing epitopes exist on RBD, and to a lesser extent NTD. This is further exemplified by the identification of RBD-specific mAbs from all five competition groups that have neutralization activity against SARS-CoV-1. Interestingly, mAbs isolated following BA.1 BTI had greater cross-neutralization of Beta compared to mAbs isolated following Delta infection. BA.1 and Beta share common mutations in RBD (K417N, E484K, N501Y), and mAbs directed against these mutated epitopes could explain the high level of cross-reactivity of mAbs from VAIN3 with Beta.

The numbers of mAbs isolated are too small to draw strong conclusions regarding differences in epitope immunodominance upon different variant exposure. However, it is clear that neutralization activity converges on similar Spike epitopes. Since the Delta and BA.1 infection waves, SARS-CoV-2 has continued to mutate. Many of the BTI mAbs isolated here lost or had greatly reduced neutralization activity against currently circulating VOCs (i.e., early 2023), including BA.2.75.2, XBB/XBB.1.5, and BQ.1.1. This pattern was also observed in sera/plasma from BTI individuals and has been reported by several other groups worldwide (65 – 68). This suggests that mAbs generated during the large Delta and BA.1 infection waves between June 2021 and March 2022 may have acted as selective pressures in driving immune escape of these VOCs, in particular selecting mutations within RBD. Indeed, BA.2.75.2 was highly prevalent in India following a large Delta wave (69). BA.2.75.2, XBB/XBB.1.5, and BQ.1.1. converge in their RBD mutational profile. BA.2.75.2, XBB/XBB.1.5, and BQ.1.1. share common mutations including R346T (within the competition Group 4 epitope) and N460K (within the competition Group 1 epitope), and XBB and BA.2.75.2 also share G446S and F486S mutations (within competition Group 3 epitope). Wang et al. demonstrated that the introduction of R346T, K444T, or N460K into BA.4/5 and R346T, V445P, or N460K into BA.2 were responsible for reduction in neutralization by many RBD-specific mAbs (65). However, the identification of mAbs belonging to several RBD competition groups that had neutralization activity against all VOCs tested suggests that multiple additional Spike mutations would be required across RBD to generate complete immune evasion of the antibody response following BTI. The mAb response was diverse in gene usage despite multiple clonal expansions being observed. Maintaining a diverse response would not only limit immune escape through selection of Spike mutations but may also represent a wide pool of B cells that could be re-activated by a diverse range of antigenically distinct Spike variants. Further studies characterizing the antibody-Spike interaction at the molecular level would provide information on how these mAbs retain cross-neutralizing activity despite high levels of Spike mutations and may help to predict future Spike escape variants.

Encouragingly, RBD-specific mAbs with cross-neutralizing activity against the most recent VOCs (BA.2.75.2, XBB/XBB.1.5, and BQ.1.1) were found within four RBD competition groups. These less frequent mAbs represent potential candidates for the next generation of antibody-based therapeutics against SARS-CoV-2 and other betacoronaviruses. Although these mAbs represent a minor component of the mAb response, understanding how to selectively boost these responses could aid in preparedness against new SARS-CoV-2 variants as they arise. Overall, infection with a VOC following two COVID-19 vaccine doses leads to production of mAbs with broad cross-neutralizing activity most likely arising through re-activation and subsequent maturation of existing Spike-specific B cells.

### Limitations

The main limitation of this study is that mAbs were studied from a single time point. Longitudinal samples were not available from the donors studied and therefore, direct comparison of BTI mAbs with mAbs present prior to infection could not be carried out. Furthermore, due to the relatively small number of mAbs cloned, we were unable to fully assess the presence and/or nature of rare variant-specific mAbs (55) or provide a more detailed assessment of mAb clonal expansions. Structural analysis of Fabs in complex with Spike would provide insight into how mAbs are able to cross-neutralize all SARS-CoV-2 variants. The conclusions from this study would benefit from the isolation of mAbs from additional vaccinated donors experiencing Delta or BA.1 infection as well as isolation of B cells using the full Spike to allow identification of mAbs against the more conserved S2 domain.

## MATERIALS AND METHODS

### Samples

SARS-CoV-2 cases were diagnosed by either reverse transcriptase PCR (RT-PCR) of respiratory samples at St Thomas’ Hospital, London, UK or by lateral flow testing. All participants were reported to be SARS-CoV-2 naïve prior to vaccination and infection and had been undergoing regular workplace testing. Participants VAIN1 and VAIN2 were infected during the UK Delta wave (11/8/21 and 23/8/21, respectively), and participant VAIN3 was infected during the UK BA.1 wave (18/12/21). Viral sequencing was not performed on these samples.

### Antigen-specific B cell sorting

Fluorescence-activated cell sorting (FACS) of cryopreserved peripheral blood mononuclear cells (PBMCs) was performed on a BD FACS Melody as previously described (5, 6). Sorting baits with a Strep2A tag (SARS-CoV-2 Wuhan S1, Delta S1, and BA.1 S1) was pre-complexed with the StrepTactin fluorophore at a 1:1 molar ratio prior to addition to cells. PBMCs were stained with live/dead (fixable Aqua Dead, Thermofisher), anti-CD3-APC/Cy7 (Biolegend), anti-CD8-APC-Cy7 (Biolegend), anti-CD14-BV510 (Biolegend), anti-CD19-PerCP-Cy5.5 (Biolegend), anti-IgM-PE (Biolegend), anti-IgD-Pacific Blue (Biolegend), anti-IgG-PeCy7 (BD), S1-StrepTactin XT DY-649 (IBA life sciences, 2-1568-050), and S1-StrepTactin XT DY-488 (IBA life sciences, 2-1562-050). Live CD3/CD8^−^CD14^−^CD19^+^IgM^−^IgD^−^IgG^+^S1^+^S1^+^ cells were sorted using a BD FACS Melody into individual wells containing RNase OUT (Invitrogen), First Strand SuperScript III buffer, dithiothreitol (DTT) and H_2_O (Invitrogen), and RNA was converted into cDNA (SuperScript III Reverse Transcriptase, Invitrogen) using random hexamers (Bioline Reagents Ltd) following the manufacturer’s protocol.

### Full-length antibody cloning and expression

The human Ab variable regions of heavy and kappa/lambda chains were PCR amplified using previously described primers and PCR conditions (32, 33, 70). PCR products were purified and cloned into human-IgG (heavy, kappa or lambda) expression plasmids (33) using the Gibson Assembly Master Mix (NEB) following the manufacturer’s protocol. Gibson assembly products were directly transfected into HEK-293T/17 cells and transformed under ampicillin selection. Ab supernatants were harvested 3 days after transfection, and IgG expression and Spike reactivity were determined using ELISA. Ab variable regions of heavy-light chain pairs that generated Spike reactive IgG were sequenced by Sanger sequencing.

Antibody heavy and light plasmids were co-transfected at a 1:1 ratio into HEK-293F cells (Thermofisher) using PEI Max (1 mg/mL, Polysciences, Inc.) at a 3:1 ratio (PEI Max:DNA). Ab supernatants were harvested 5 days following transfection, filtered, and purified using protein G affinity chromatography following the manufacturer’s protocol (GE Healthcare).

### Pseudovirus production

HEK293T/17 cells were seeded the day prior on 10 cm dishes at a density of 7 × 10^5^ cells/mL in Dulbecco's Modified Eagle Medium (DMEM) with 10% fetal bovine serum (FBS) and 1% Pen/Strep. Cells were co-transfected using 90 µg PEI-Max (1 mg/mL, Polysciences) with 15 µg HIV-luciferase plasmid, 10 µg HIV 8.91 gag/pol plasmid (71), and 5 µg SARS-CoV-2 Spike protein plasmid. Transfected cells were incubated for 72 h at 37°C, and virus was harvested, sterile filtered, and stored at −80°C until required. Mutations present in each variant Spike are shown in Table S4.

### Neutralization assays

Serial dilutions of sera or mAb in DMEM, supplemented with 10% FBS and 1% Pen/Strep, were incubated in a 96-well plate, with HIV-1 virus pseudotyped with SARS-CoV-2 wild-type or variant Spikes for 1 h at 37°C. HeLa cells stably expressing the human ACE2 receptor were then added at a density of 4 × 10^5^ cells/mL to all wells and incubated for 72 h at 37°C. Levels of infection were measured with the Bright-Glo luciferase kit (Promega) on a Victor X3 multilabel reader (Perkin Elmer). Duplicate measurements were used to calculate IC_50_ and ID_50_.

### ELISA (Spike, RBD, NTD, or S1)

Ninety-six-well plates (Corning, 3690) were coated with Spike, S1, NTD, or RBD at 3 µg/mL in phosphate buffered saline (PBS) overnight at 4°C. The plates were washed (five times with PBS/0.05% Tween-20, PBS-T) and blocked with blocking buffer (5% skimmed milk in PBS-T) for 1 h at room temperature. Serial dilutions of mAb or supernatant in blocking buffer were added and incubated for 2 h at room temperature. Plates were washed (five times with PBS-T), and secondary antibody was added and incubated for 1 h at room temperature. IgG was detected using goat-anti-human-Fc-AP (alkaline phosphatase) (1:1,000) (Jackson: 109-055-098). Plates were washed (five times with PBS-T) and developed with AP substrate (Sigma) and read at 405 nM.

### Competition ELISA

*F*(ab′)_2_ of previously characterized mAbs were produced by IdeS digestion of IgG as described previously (5). Ninety-six-well plates (Corning, 3690) were coated with WT Spike at 3 µg/mL overnight at 4°C. Plates were washed and blocked as described above. Serial dilutions (five-fold) of *F*(ab′)_2_, starting at 100-fold molar excess of the EC_80_ of Spike binding were added to the plate and incubated for 1 h at room temperature. Plates were washed (five times with PBS-T), and competing IgG was added at the EC_80_ of Spike binding and incubated for 1 h at room temperature. Plates were washed (five times with PBS-T), and goat-anti-human-Fc-AP (alkaline phosphatase) (1:1,000) (Jackson: 109-055-098) was added and incubated for 1 h at room temperature. The plates were washed a final time (five times with PBS-T), and the plate was allowed to develop by addition of AP substrate (Sigma). Optical density at 405 nM was measured in 5 min intervals. Percentage competition was calculated using the equation below and competition group clusters were arranged by hand according to binding epitope.


$$\%\;IgG\;competition=100\times \left(1-\frac{OD_{405}\;of\;F{\left(ab^{\prime }\right)}_{2}\;sample\;well-mean\;OD_{405}\;of\;background\;}{OD_{405}\;of\;IgG\;only\;well-mean\;OD_{405}\;of\;background}\right)$$

### ACE2 competition measured by flow cytometry

Fluorescent probe was prepared by mixing 3.5 M excess of Streptavidin-APC (Thermofisher Scientific, S32362) with biotinylated SARS-CoV-2 spike and incubating for 1 h on ice. Purified mAb was mixed with APC conjugated Spike in a molar ratio of 4:1 in FACS buffer (2% FBS in PBS) on ice for 1 h. HeLa-ACE2 cells were washed once with PBS and detached using 5 mM EDTA in PBS. Cells were washed and resuspended in FACS buffer before adding 5 × 10^5^ cells to each mAb-Spike complex. Cells were incubated on ice for 30 min. HeLa-ACE2 cells alone and with SARS-CoV-2 Spike only were used as background and positive controls, respectively. The geometric mean fluorescence of APC was measured from the gate of singlet cells. ACE2 binding inhibition was calculated with the following equation:


$$\%ACE2\;binding\;inhibition=100\times \left(1-\frac{sample\;geometric\;mean-background\;geometric\;mean}{positive\;control\;geometric\;mean-background\;geometric\;mean}\right)$$

### Sequence analysis of monoclonal antibodies

Heavy and light chain sequences of SARS-CoV-2-specific mAbs were examined using IMGT/V-quest (http://www.imgt.org/IMGT_vquest/vquest) to identify germline usage, percentage of SHM and CDR region lengths. Five amino acids or 15 nucleotides were truncated from the start and end of the sequences to remove variation introduced from the use of a mixture of forward cloning primers. D’Agostino and Pearson tests were performed to determine normality. Based on the result, a Kruskal-Wallis test with Dunn’s multiple comparison post hoc test was performed. Two-sided binomial test was performed in excel. Significance is defined as *P < 0.0332, **P < 0.0021, ***P < 0.0002, and ****P > 0.0001.

## Data Availability

All data needed to evaluate the conclusions in the paper are present in the paper and/or the supplemental material. The antibody sequences generated during this study are available at GenBank (accession numbers OR427042 - OR427279).

## SUPPLEMENTAL MATERIAL

Supplemental material mbio.01206-23-s0001.pdf Supplemental figures and tables.

ASM does not own the copyrights to Supplemental Material that may be linked to, or accessed through, an article. The authors have granted ASM a non-exclusive, world-wide license to publish the Supplemental Material files. Please contact the corresponding author directly for reuse.
