# Supplementary material for: Broad and potent neutralizing antibodies are elicited in vaccinated individuals following Delta/BA.1 breakthrough infection
Source: mBio. 2023 Sep 25;14(5):e01206-23. doi: 10.1128/mbio.01206-23 (PMC10653880; doi:10.1128/mbio.01206-23)
Supplement: Supplemental material — Supplemental figures and tables. [file mbio.01206-23-s0001.pdf]

Broad and potent neutralizing antibodies are elicited in vaccinated individuals following Delta/BA.1 breakthrough infection

Jeffrey Seow[1]*, Zayed A. Shalim[1]*, Carl Graham[1], Simon Kimuda[1], Aswin Pillai[1], Thomas Lechmere[1], Ashwini Kurshan[1], Atika M. Khimji[1], Luke B. Snell[1,2], Gaia Nebbia[2], Christine Mant[1,3], Anele Waters[4], Julie Fox[1,4], Michael H. Malim[1], Katie J. Doores[1]#

[1] Department of Infectious Diseases, School of Immunology & Microbial Sciences, King's College London, London, UK.

[2] Centre for Clinical Infection and Diagnostics Research, Department of Infectious Diseases, Guy's and St Thomas' NHS Foundation Trust, London, UK.

[3] Infectious Diseases Biobank, Department of Infectious Diseases, School of Immunology and Microbial Sciences, King's College London, London, UK.

[4] Harrison Wing, Guys and St Thomas' NHS Trust, London, UK.

* These authors contributed equally

# Corresponding author: katie.doores@kcl.ac.uk

Figure S1: Sorting strategy for isolation of S1-reactive B cells following infection in vaccinated individuals. Strategy to isolate SARS-CoV-2 S1-specific IgG+ B cells. Example sorting for donor VAIN1. Live CD3/CD8$^-$CD14$^-$CD19$^+$IgM$^-$IgD$^-$IgG$^+$S1$^+$S1$^+$ cells were sorted into individual wells. The heavy and light chains were reverse transcribed and amplified using nested PCR with gene specific primers[1-3]. Related to Figure 1.

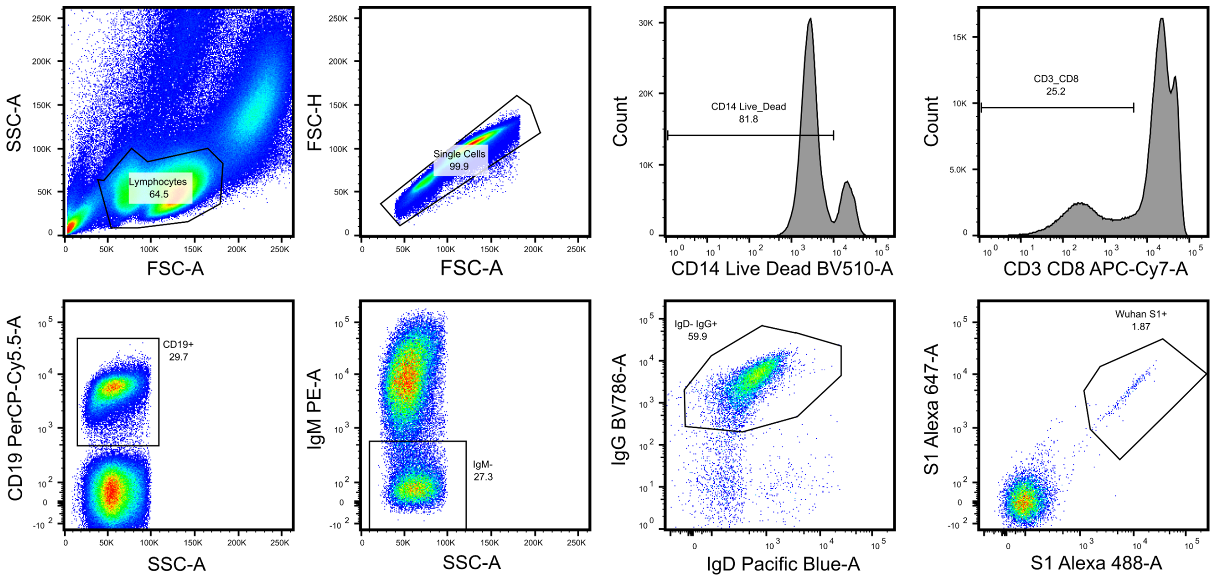

Figure S2: Plasma cross-neutralizing activity for donors VAIN1, VAIN2 and VAIN3. Neutralization was tested using HIV-1 viral particles pseudotyped with Spike of Wuhan-1 (WT), beta, delta, BA.1, BA.2 and BA.4/5. Blood samples for plasma isolation were collected 15, 87 and 26 days post infection, respectively. Related to Figure 1.

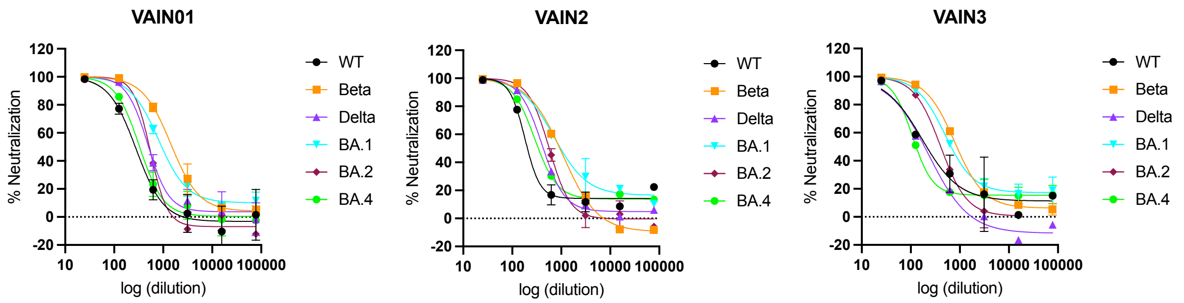

Figure S3: Comparison between mAbs isolated from VAIN1, VAIN2 and VAIN3. A) Truncated violin plot comparing the level of nucleotide mutation from germline for V_H and V_L between mAbs isolated from VAIN1, VAIN2 and VAIN3. **B)** Truncated violin plot comparing the level of nucleotide mutation from germline for V_H and V_L between B cells selected using WT S1, Delta S1 or BA.1 S1. **C)** Plot comparing the level of nucleotide mutation between WT and VOC selected B cells for donors VAIN1, VAIN2 and VAIN3, respectively. D'Agostino and Pearson tests were performed to determine normality. Based on the result, a Kruskal-Wallis test with Dunn's multiple comparison post hoc test was performed. *p < 0.0332, **p < 0.0021, ***p < 0.0002, and ****<0.0001. **D)** Correlation of V_H and V_L % divergence from germline at the nucleotide level and amino acid level for BTI mAbs. (Spearman correlation, two-tailed, r). A linear regression was used to calculate the goodness of fit (r^2). **E)** Distribution of CDRH3 lengths for mAbs isolated following BTI and representative naive B cell repertoire[4]. Error bars represent the standard deviation between donors used in the analysis (n = 3 for BTI mAbs and n = 10 for naive repertoire). A bimodal distribution of CDRH3 length is observed for SARS-CoV-2 Spike reactive mAbs. Related to Figure 2.

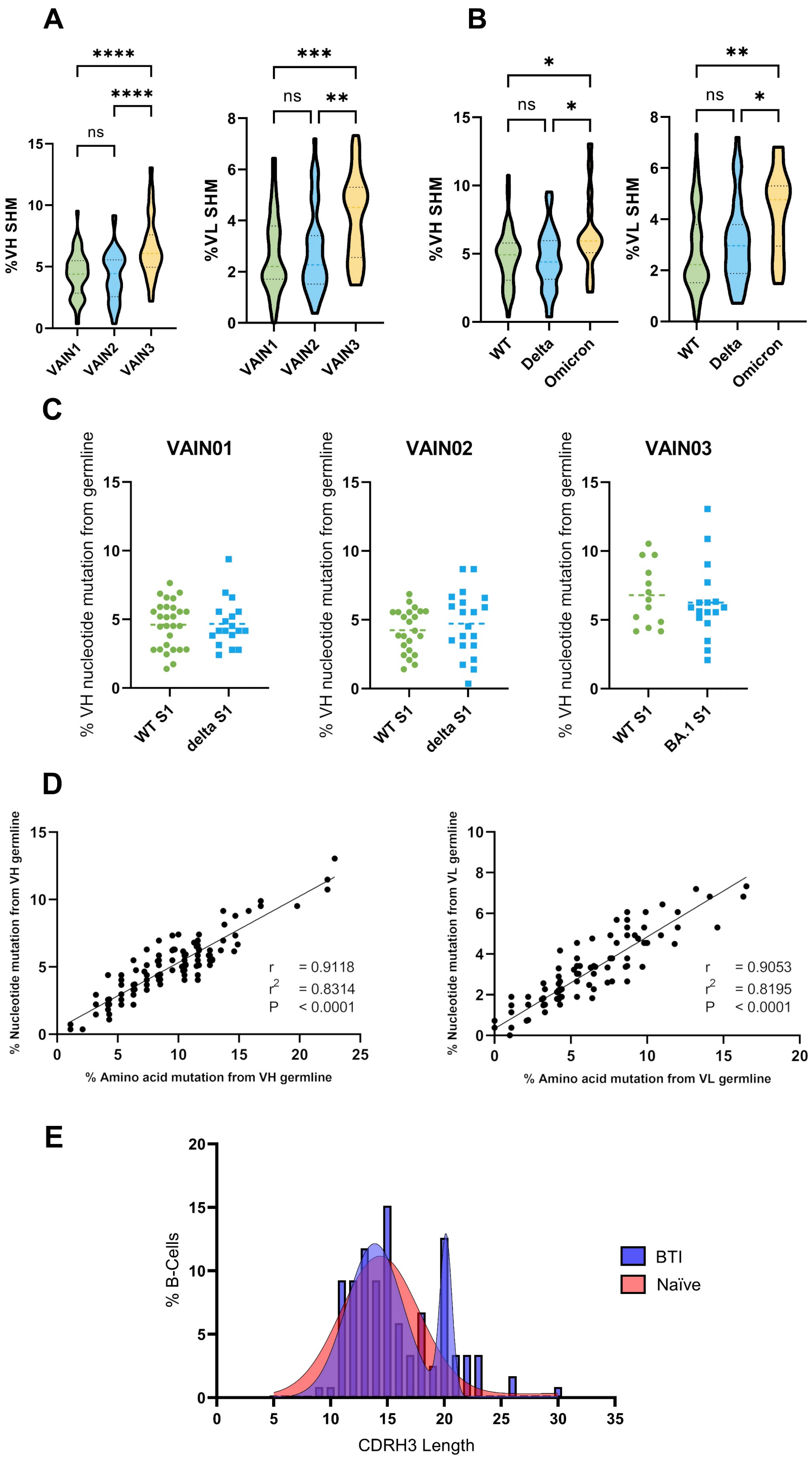
A
B
****
****
ns
%VH SHM
VAIN1
VAIN2
VAIN3
***
ns
**
%VL SHM
*
ns
*
WT
Delta
Omicron
**
ns
*
C
VAIN01
VAIN02
VAIN03
% VH nucleotide mutation from germline
WT S1
delta S1
BA.1 S1
D
% Nucleotide mutation from VH germline
% Amino acid mutation from VH germline
r = 0.9118
r2 = 0.8314
P < 0.0001
% Nucleotide mutation from VL germline
% Amino acid mutation from VL germline
r = 0.9053
r2 = 0.8195
P < 0.0001
E
% B-Cells
CDRH3 Length
BTI
Naïve

Figure S4: Correlation between WT and BTI VOC neutralization potency. A) Correlation between IC_{50} values against WT and delta (VAIN1 and VAIN2 mAbs) or against WT and BA.1 (VAIN3 mAbs). Delta correlation is shown in purple and BA.1 correlation is shown in blue. **B)** Correlation between IC_{50} values and level of somatic hypermutation for mAbs isolated from VAIN1, VAIN2 and VAIN3. Dotted line represents the highest mAb concentration tested. (Spearman correlation, r. A linear regression was used to calculate the goodness of fit, r^2). Significant correlations are highlighted in bold in the table. Related to Figures 2 and 3.

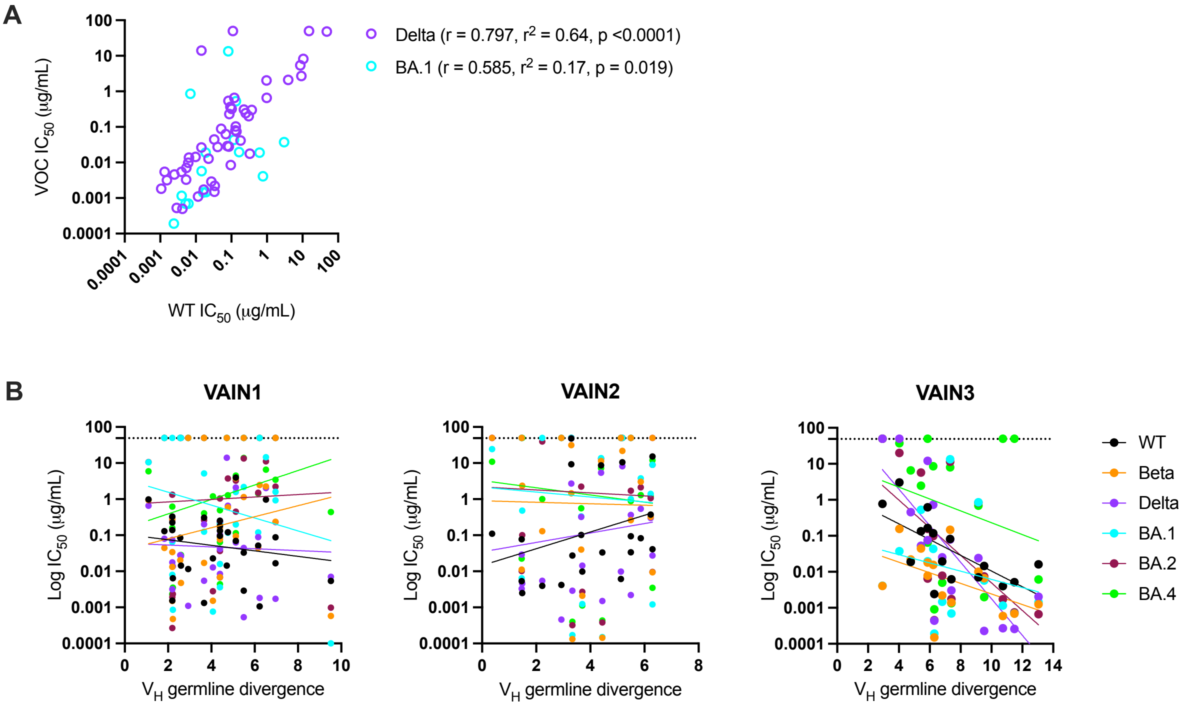

	WT	Beta	Delta	BA.1	BA.2	BA.4
**VAIN1**	r^2: 0.027<br>r: -0.21<br>*p* = ns	r^2: 0.037<br>r: 0.359<br>*p* = ns	r^2: 0.0022<br>r: -0.022<br>*p* = ns	r^2: 0.042<br>r: -0.073<br>*p* = ns	r^2: 0.0015<br>r: 0.14<br>*p* = ns	r^2: 0.073<br>r: 0.289<br>*p* = ns
**VAIN2**	r^2: 0.098<br>r: 0.346<br>*p* = ns	r^2:0.0003<br>r: -0.103<br>*p* = ns	r^2: 0.021<br>r: 0.166<br>*p* = ns	r^2:0.0039<br>r: -0.192<br>*p* = ns	r^2: 0.0016<br>r: -0.224<br>*p* = ns	r^2: 0.009<br>r: -0.166<br>*p* = ns
**VAIN3**	**r^2:0.44**<br>**r: -0.752**<br>***p* = 0.012**	**r^2: 0.23**<br>**r: -0.593**<br>***p* = 0.017**	**r^2: 0.61**<br>**r: -0.841**<br>***p* <0.0001**	r^2:0.060<br>r: -0.380<br>*p* = ns	**r^2: 0.38**<br>**r: -0.661**<br>***p* = 0.0065**	r^2:0.061<br>r: -0.235<br>*p* = ns

Figure S5: RBD- and NTD-specific mAbs form multiple competition groups. Competition for **A)** RBD-specific mAbs and **B)** NTD-specific mAbs. Inhibition of IgG binding to SARS-CoV-2 Spike by F(ab)$_2$’ fragments was measured. The percentage competition was calculated using the reduction in IgG binding in the presence of F(ab’)$_2$ (at 100-molar excess of the IC$_{80}$) as a percentage of the maximum IgG binding in the absence of F(ab’)$_2$. Competition groups clusters were arranged by hand according to binding epitopes. Experiments were performed in duplicate. Competition <25% is white. Grey boxes indicate competition not tested. Competition groups are colour-coded according to the key. Related to Figures 4 and 5.

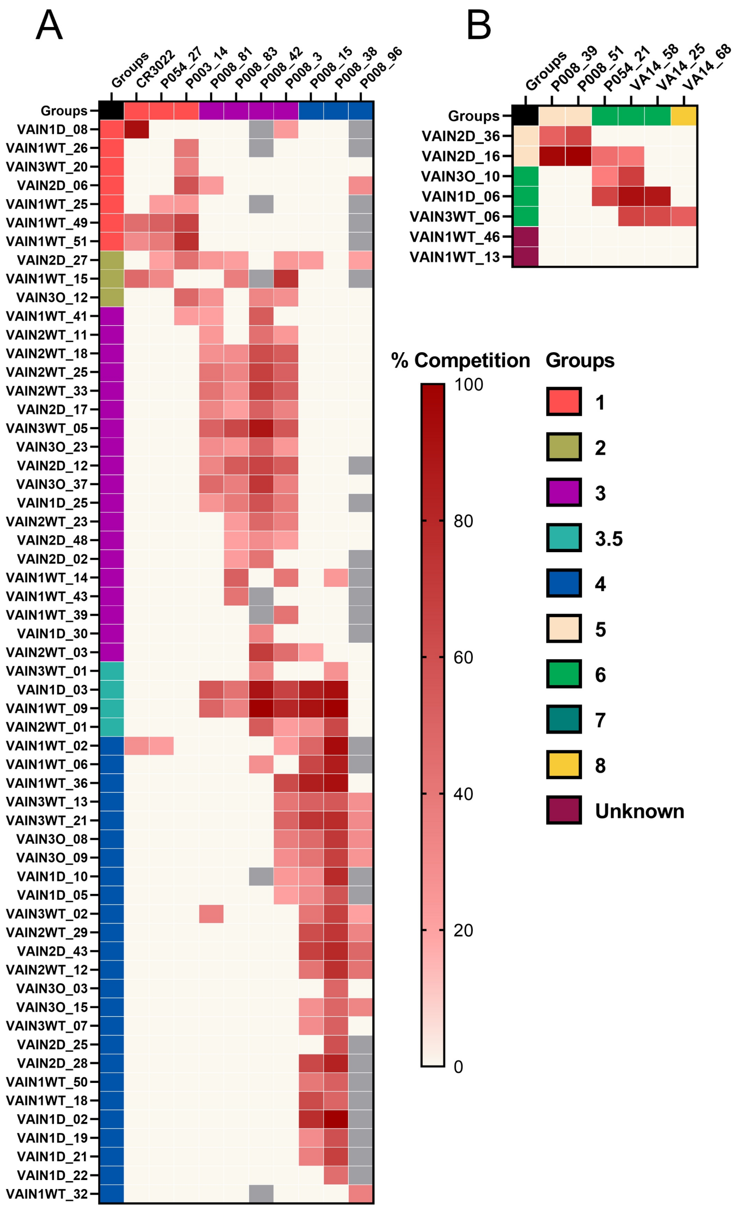

Figure S6: RBD-specific mAb neutralization geometric mean IC_{50} against SARS-CoV-2 VOCs by competition group. Dotted line represents the highest mAb concentration tested. The horizontal line shows the geometric mean IC_{50} for each RBD competition group. Related to Figure 4.

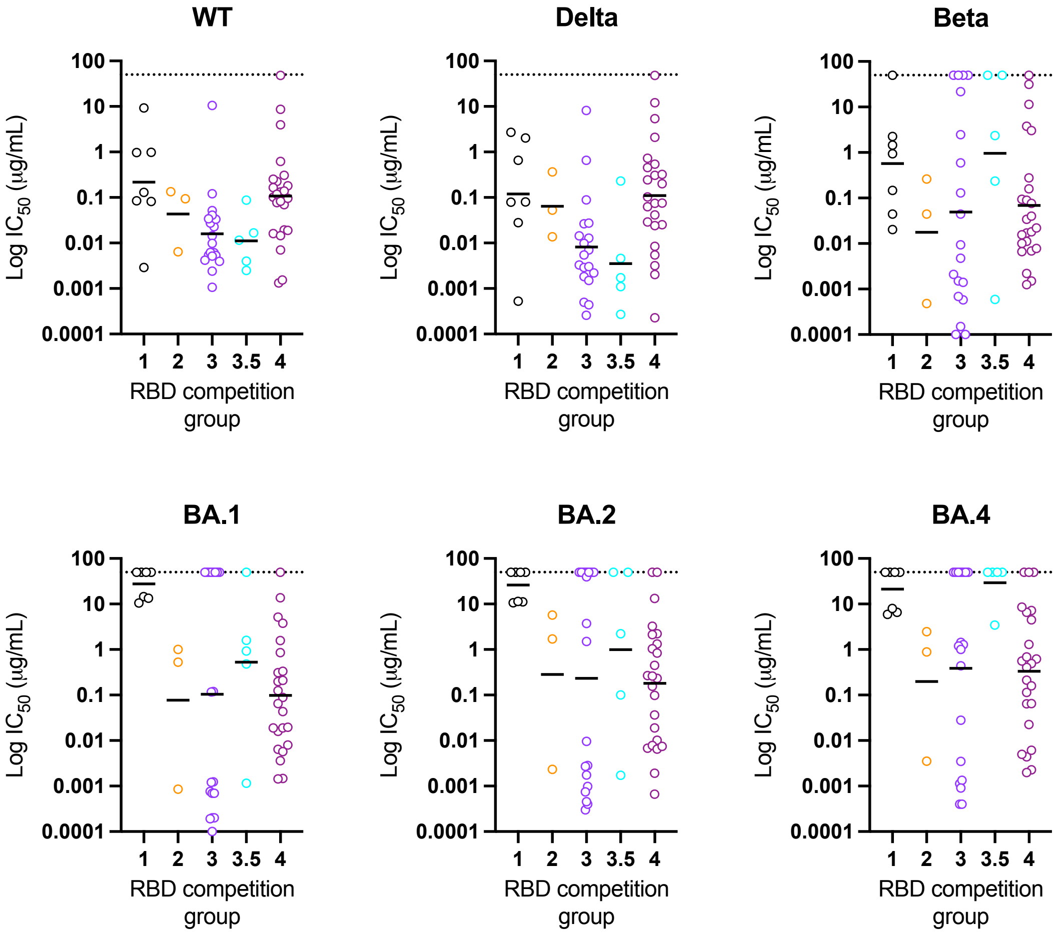

Figure S7: VAIN1, VAIN2 and VAIN3 plasma neutralization against BA.2.75.2, XBB and BQ.1.1. Related to Figure 6.

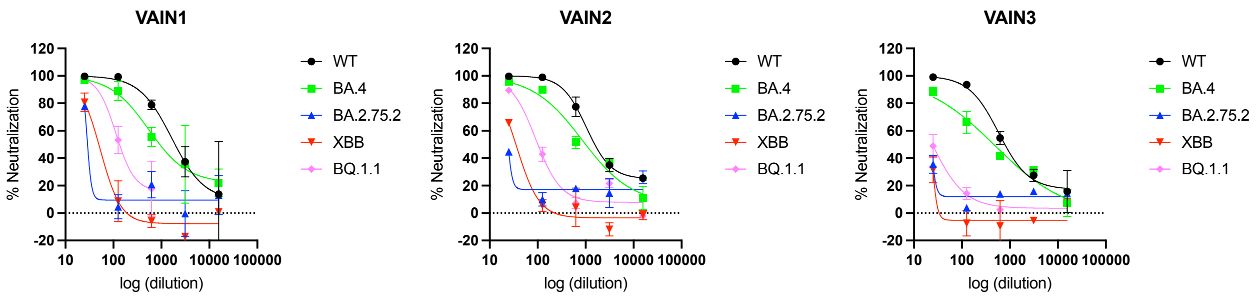

Supplementary Table 1: VAIN1, VAIN2 and VAIN3 donor information. Dates for vaccination and SARS-CoV-2 infection and the days between these events and blood donation. Related to Figure 1.

	VAIN1	VAIN2	VAIN3
Gender	Female	Male	Female
Ethnicity	White	White	White
Age	na	42	32
1st Vaccine (Vaccine type)	12/01/2021 (Pfizer mRNA:BNT162b2)	21/01/2021 (Pfizer mRNA:BNT162b2	21/05/2021 (Pfizer mRNA:BNT162b2)
2nd Vaccine (Vaccine type)	02/03/2021 (Pfizer mRNA:BNT162b2	03/03/2021 (Pfizer mRNA:BNT162b2)	01/07/2021 (Pfizer mRNA:BNT162b2)
Infection date and SARS-CoV-2 strain	11/08/2021 Presumed Delta (B.1.617.2)	23/08/2021 Presumed Delta (B.1.617.2)	18/12/2021 Presumed Omicron (BA.1)
PBMC sample date	26/08/2021	18/11/2021	13/01/2022
Days post 2nd vaccine	163	173	170
Days post infection	15	87	26

Supplementary Table 2: Clonally related mAbs isolated from VAIN1, VAIN2 and VAIN3.

Donor	Name	Heavy V gene	Heavy CDR3 AA	Heavy CDR3 length	Light V gene	Light CDR3 AA	Light CDR3 length
VAIN1	V1D_10	IGHV1-3	CARGPEMAIVDYFDYW	16	IGKV1-5	CQQYNGYPWTF	11
	V1D_22	IGHV1-3	CARSGGGFLVDYMDVW	16	IGKV1-5	CQQYHGYPWTF	11
VAIN2	V2WT_30	IGHV3-30	CARDGKTINMVRGVISGAFDIW	22	IGKV1-33	CQQYDNLPFTF	11
	V2D_42	IGHV3-30	CARDGRTINMVRGVISGAFDIW	22	IGKV1-33	CLQYDILPFTF	11
	V2WT_11	IGHV3-30	CARDGRTITMVRGVISGAFDIW	22	IGKV1-33	CQQYDNLPFTF	11
	V2WT_23	IGHV3-30	CARDGRTITMVRGVISGAFDIW	22	IGKV1-33	CQQYDNLPSTF	11
	V2D_48	IGHV3-30	CARDGTMAPLVPGIMSPAFDIW	22	IGKV1-33	CQQYDNLPPTF	11
	V2WT_33	IGHV3-53	CARDLELAGALDVW	14	IGKV1-9	CQQINSNPPVTF	12
	V2WT_25	IGHV3-53	CARDLELAGGLDIW	14	IGKV1-9	CQQLNSYPPVTF	12
	V2WT_2	IGHV4-59	CARDLAYGEYEGWFDPW	17	IGKV1-12	CQQAYSFPYTF	11
	V2D_34	IGHV4-59	CARDLTYGEYEGWFDPW	17	IGKV1-12	CQQAHSFPYTF	11
VAIN3	V3O_21	IGHV1-69	CAIVFGDQSEFDSW	14	IGKV3-11	CQFRSNWPPYTF	12
	V3O_28	IGHV1-69	CAIVFGDQSEFDSW	14	IGKV3-11	CQFRSNWPPYTF	12
	V3WT_11	IGHV3-15	CTTDIYILGVMIEHDAFDSW	20	IGKV1-39	CQQTYTTPAPSF	12
	V3O_27	IGHV3-15	CTTDLYILGVVIEHDAFDIW	20	IGKV1-39	CQQTYFAPALTF	12
	V3O_37	IGHV3-53	CARDFGEMYFDYW	13	IGKV3-20	CQQYGNSPRTF	11
	V3WT_18	IGHV3-53	CARDYGEMYFDFW	13	IGKV3-20	CQQYGGSPRTF	11
	V3O_26	IGHV3-66	CARGFGDQYFDLW	13	IGKV1-39	CQQSYSYPLTF	11
	V3WT_5	IGHV3-66	CARGIGDQYFDLW	13	IGKV1-39	CQQSYSSPLTF	11
	V3WT_9	IGHV3-7	CARGGGHPWYYSGSGSYPPLPKADLDYW	28	IGLV1-44	CVAWDDSLKGSWVF	14
	V3WT_6	IGHV3-7	CARGGGHPWYYSSSGNFPPLPKADLDYW	28	IGLV1-44	CVAWDDSLKGSWVF	14
	V3O_23	IGHV4-34	CARACSGGNCYPRPFDYW	18	IGKV1-17	CLQHNSYPWTF	11
	V3WT_1	IGHV4-34	CARGCSGGICYPKPFDFW	18	IGKV1-17	CLQHNSLPWTF	11

Related to Figure 2.

Supplementary Table 3: Neutralization properties of large-scale expressed nAbs. mAb are listed based on competition group**.** IC_{50} values are reported in µg/mL. X indicates neutralization not tested. Related to Figures 1-6.

Name	IC50 WT	IC50 Beta	IC50 Delta	IC50 Omicron	IC50 BA2	IC50 BA4	IC50 D614G	IC50 BA.4	IC50 XBB	IC50 BQ.1.1	IC50 BA.2.75.2	IC50 XBB.1.5	ACE2 Comp	Specificity	Comp Group	Heavy chain V Gene	Heavy chain CDR3 AA	Light chain V gene	Light chain CDR3 AA
VAIN1WT_49	0.977	2.243	2.022	14.508	11.144	6.592	6.317	10.786	>50	>50	2.016	>50	24	RBD	1	IGHV4-39	CARHLEEILPKGVNWFDPW	IGKV1-39	CQQSYATLPYTF
VAIN1D_08	0.003	>50	0.001	>50	>50	>50	0.001	>50	>50	>50	>50	>50	0	RBD	1	IGHV1-69	CARGPPLTRGVARAGQAFDYW	IGKV4-1	CQQYYSSSITF
VAIN1WT_51	0.980	0.940	0.654	10.558	10.620	5.946	3.323	4.205	10.438	17.792	15.575	X	0	RBD	1	IGHV3-30	CATDSSDPWQNYFDYW	IGKV1-39	CQQSYSTFEYTF
VAIN1WT_25	0.130	0.044	0.080	>50	>50	>50	X	X	X	X	X	X	99	RBD	1	IGHV5-10-1	CARGPSYYHTGRMGDVW	IGKV1-33	CLQYDSLLGTF
VAIN1WT_26	0.084	0.020	0.028	>50	>50	>50	X	X	X	X	X	X	98	RBD	1	IGHV1-69	CACGGYYDTSGYYALDFDSW	IGKV1-5	CQQFNSYSRTF
VAIN2D_06	9.274	1.457	2.700	>50	>50	>50	X	X	X	X	X	X	92	RBD	1	IGHV3-23	CAEPRTGWYVGFDYW	IGLV6-57	CQSYDSSNHYVF
VAIN3WT_20	0.082	0.147	0.079	13.395	11.448	7.948	X	X	X	X	X	X	94	RBD	1	IGHV3-11	CARQKWLRGDFDSW	IGLV6-57	CQSYDSRNWMF
VAIN2D_27	0.093	0.262	0.365	1.002	1.700	0.891	0.235	1.251	0.495	0.436	0.401	3.159	78	RBD	2	IGHV3-30	CARDWGRLVTWFDNW	IGKV1-39	CQQSYSTPPWTF
VAIN3O_12	0.135	0.044	0.053	0.529	5.745	2.475	0.008	1.849	2.176	3.916	2.337	3.076	86	RBD	2	IGHV4-61	CARDLWYYDRSGHYDSDAFDVW	IGLV1-40	CQSYDSSLTALF
VAIN1WT_15	0.006	0.0005	0.014	0.001	0.002	0.004	0.023	0.001	>50	9.567	>50	>50	100	RBD	2	IGHV3-53	CARDLVVYGMDVW	IGKV1-9	CQQLNSDAISF
VAIN2D_12	0.010	0.002	0.014	0.001	0.003	0.001	0.008	0.003	0.004	0.004	0.005	0.002	99	RBD	3	IGHV3-66	CARAYGDRYYFDYW	IGKV1-5	CQHYGAF
VAIN2D_17	0.041	0.009	0.027	0.001	0.010	0.003	0.061	0.008	0.015	0.011	0.015	0.003	99	RBD	3	IGHV3-53	CARDFYRQSGDYPHDFW	IGKV1-33	CHQYDNLPRTF
VAIN3O_37	0.002	0.0002	0.0004	0.0002	0.0005	0.001	0.004	0.001	1.347	0.051	0.474	0.147	97	RBD	3	IGHV3-53	CARDFGEMYFDYW	IGKV3-20	CQQYGNSPRTF
VAIN1WT_14	0.014	0.005	0.027	>50	>50	0.028	0.005	0.028	0.250	0.281	0.479	0.106	97	RBD	3	IGHV3-53	CARLYNHRRGMDVW	IGKV1-33	CQQYDNLPITF
VAIN2WT_33	0.033	0.0001	0.002	0.0002	0.0004	0.0004	0.005	0.001	>50	0.103	1.699	1.211	94	RBD	3	IGHV3-53	CARDLELAGALDVW	IGKV1-9	CQQINSNPPVTF
VAIN2WT_25	0.028	0.0001	0.003	0.0002	0.0003	0.0004	0.013	0.002	>50	0.219	13.184	>50	93	RBD	3	IGHV3-53	CARDLELAGGLDIW	IGKV1-9	CQQLNSYPPVTF
VAIN3WT_05	0.006	0.001	0.003	0.001	0.002	0.001	0.002	0.001	>50	2.527	2.477	>50	99	RBD	3	IGHV3-66	CARGIGDQYFDLW	IGKV1-39	CQQSYSSPLTF
VAIN1D_30	0.005	0.001	0.007	0.0001	0.001	0.438	0.006	0.271	>50	1.865	>50	>50	98	RBD	3	IGHV3-64	CVKGGIQLWPTGDSW	IGKV1-5	CQQYKSYPWTF
VAIN3O_23	0.005	0.001	0.0003	0.001	0.001	>50	0.001	>50	>50	>50	>50	>50	97	RBD	3	IGHV4-34	CARACSGGNCYPRPFDYW	IGKV1-17	CLQHNSYPWTF
VAIN2WT_23	0.004	>50	0.0005	>50	>50	>50	0.004	>50	>50	>50	>50	>50	89	RBD	3	IGHV3-30	CARDGRTITMVRGVISGAFDIW	IGKV1-33	CQQYDNLPSTF
VAIN2D_48	0.034	>50	0.002	>50	>50	>50	0.034	>50	>50	>50	>50	>50	90	RBD	3	IGHV3-30	CARDGTMAPLVPGIMSPAFDIW	IGKV1-33	CQQYDNLPPTF
VAIN1D_25	0.023	0.002	0.013	0.001	0.003	1.191	0.005	0.756	>50	>50	>50	X	99	RBD	3	IGHV3-53	CARDLAPVGLMDVW	IGKV1-27	CQNYNSDPPWTF
VAIN1WT_43	0.121	0.594	0.660	0.117	3.754	1.287	2.210	6.019	10.646	34.975	13.252	X	0	RBD	3	IGHV3-33	CARDEGAVVTHMDYW	IGKV1-39	CQQSYNTPPWTF
VAIN1WT_39	0.001	2.460	0.002	>50	>50	>50	X	X	X	X	X	X	99	RBD	3	IGHV5-10-1	CARQSGDYFDYLLAYFDLW	IGKV3-20	CQQYSSSPPGYTF
VAIN2WT_11	0.005	>50	0.003	>50	>50	>50	X	X	X	X	X	X	81	RBD	3	IGHV3-30	CARDGRTITMVRGVISGAFDIW	IGKV1-33	CQQYDNLPFTF
VAIN2WT_18	10.565	21.841	8.213	>50	>50	>50	X	X	X	X	X	X	18	RBD	3	IGHV3-53	CARVLPYGDNYFDFW	IGKV3-11	CQQLFTF
VAIN2D_02	0.004	0.130	0.005	>50	39.604	1.000	X	X	X	X	X	X	99	RBD	3	IGHV3-66	CARNVWDDAFDLW	IGKV1-9	CQLLNSYPPGTF
VAIN1WT_41	0.051	0.044	0.089	0.120	1.498	1.431	X	X	X	X	X	X	95	RBD	3	IGHV4-39	CARTAPYYYDRSGYYQKEEYFQRW	IGKV1-5	CQQYNNYPRTF
VAIN2WT_03	0.006	>50	0.010	>50	>50	>50	X	X	X	X	X	X	0	RBD	3	IGHV3-30-3	CARDGTMAPLVPGIMSPAFDIW	IGKV1-33	CQQYDNLPPTF
VAIN3WT_01	0.004	0.001	0.0003	0.001	0.002	>50	0.002	>50	>50	>50	>50	>50	0	RBD	3.5	IGHV4-34	CARGCSGGICYPKPFDFW	IGKV1-17	CLQHNSLPWTF
VAIN2WT_01	0.002	2.352	0.005	0.482	0.101	>50	0.001	>50	>50	31.732	6.108	X	99	RBD	3.5	IGHV3-53	CARESYAVATIGKEYRMDVW	IGKV3-11	CQQRSNWPWGF
VAIN1WT_09	0.012	>50	0.001	>50	>50	>50	X	X	X	X	X	X	76	RBD	3.5	IGHV1-8	CARGGRYCDITSCYSRGWLDPW	IGLV1-51	CGTWDAGLSVVF
VAIN1D_03	0.017	>50	0.002	1.587	>50	>50	X	X	X	X	X	X	0	RBD	3.5	IGHV1-2	CARDQSFSMVRGDTDHW	IGKV1-39	CQQSYRTPALSF
VAIN1WT_02	0.088	0.236	0.230	0.923	2.235	3.429	X	X	X	X	X	X	57	RBD	3.5	IGHV1-46	CARAGVAPDHSHPFDPW	IGKV1-13	CQQFNTYLSITF
VAIN1WT_06	0.225	0.276	0.306	0.310	1.326	0.620	2.334	4.325	1.289	4.834	7.452	1.551	72	RBD	4	IGHV1-18	CARGPWGYCSGTSCYYWTPDNPAYSYHGMDVW	IGKV2-24	CMQATQFPHTF
VAIN1WT_18	0.070	0.094	0.063	0.198	0.447	0.114	0.180	0.613	0.288	0.538	0.539	0.678	0	RBD	4	IGHV5-51	CARQFCGGGCHFDYW	IGKV1-5	CQQYNSYSRTF
VAIN1WT_32	0.133	0.090	0.103	0.089	0.233	0.212	0.265	0.261	0.713	2.042	2.976	0.843	28	RBD	4	IGHV5-51	CARSDTSNYFDYW	IGKV1-5	CQQYNGYSF
VAIN1WT_50	0.251	0.160	0.245	0.210	1.036	0.494	2.340	1.597	2.329	3.862	1.907	2.044	0	RBD	4	IGHV1-69	CAKGGGYSYGHHYNWFFDVW	IGKV4-1	CQQYFSTPPWTF
VAIN1D_19	0.139	0.034	0.075	0.065	0.268	0.160	0.182	0.067	0.142	1.392	1.093	0.128	4	RBD	4	IGHV5-51	CARTLQTNWLDHW	IGKV1-5	CQQFNTYWTF
VAIN2WT_29	0.102	0.040	0.320	0.332	2.220	0.557	0.102	0.557	4.580	4.338	1.787	1.244	18	RBD	4	IGHV5-51	CARRTSSAGLPCLDVW	IGKV1-39	CQQSFSTLALTF
VAIN2D_28	0.083	3.039	0.540	3.786	2.133	1.300	5.469	2.688	0.744	1.007	2.138	2.637	60	RBD	4	IGHV3-48	CARDTGFWSGHYPAQFDYW	IGKV1-33	CQQYNTPPPVTF
VAIN2D_25	0.078	0.011	0.029	0.006	0.010	0.023	0.550	0.153	0.278	>50	0.183	1.016	54	RBD	4	IGHV5-51	CARRDSGSYSGYFDLW	IGLV1-44	CAAWDDSLNGVVF
VAIN3O_09	0.016	0.001	0.002	0.001	0.001	0.006	0.006	0.004	0.038	>50	0.034	0.028	97	RBD	4	IGHV4-39	CTRMAVQWGWYGAIDYW	IGLV3-21	CQVWESIYDLPWVF
VAIN3O_15	0.015	0.007	0.0002	0.006	0.007	0.002	0.013	0.001	0.041	>50	0.009	0.049	0	RBD	4	IGHV4-31	CARGIPDSAVNSW	IGLV1-40	CQSYDSSMSGPVF
VAIN1D_05	0.181	0.077	0.042	0.016	0.037	0.064	0.144	0.229	0.766	>50	0.233	0.646	0	RBD	4	IGHV1-69	CARDMIEAPLYGMDVW	IGKV1-39	CQQSYRTPALSF
VAIN1D_22	0.096	0.007	0.008	0.004	0.007	0.004	0.017	0.003	>50	>50	1.785	>50	0	RBD	4	IGHV1-3	CARSGGGFLVDYMDVW	IGKV1-5	CQQYHGYPWTF
VAIN3WT_07	0.019	0.002	0.025	0.001	0.008	0.005	0.001	0.001	>50	>50	0.093	>50	96	RBD	4	IGHV3-15	CVTDLFVQGVILEHDAFDIW	IGKV1-39	CQQCYITPAPTF
VAIN3O_03	0.164	0.008	0.075	0.020	0.007	0.065	0.033	0.030	>50	>50	0.240	>50	0	RBD	4	IGHV3-9	CVKEKIRLREGEGMDVW	IGLV3-21	CQVWESSSDHVVF
VAIN3WT_13	0.007	0.010	0.024	0.857	0.834	0.688	0.004	0.272	>50	>50	>50	>50	95	RBD	4	IGHV1-18	CARDSSVAGSLDYW	IGLV2-14	CSSYTRTIYIF
VAIN1D_10	0.002	0.001	0.003	0.008	0.002	0.002	0.001	0.0003	>50	>50	>50	>50	9	RBD	4	IGHV1-3	CARGPEMAIVDYFDYW	IGKV1-5	CQQYNGYPWTF
VAIN3WT_02	0.619	0.018	12.037	0.019	0.156	>50	0.085	>50	>50	>50	0.002	>50	0	RBD	4	IGHV1-46	CARSHSVTLAGVDYW	IGLV7-43	CLLYYEGAWVF
VAIN2D_43	48.000	31.281	48.000	>50	>50	>50	48.000	>50	>50	>50	>50	>50	19	RBD	4	IGHV3-30	CAKDRSWFGEFQDDNYYYAMDVW	IGKV1-33	CQQYDNLPLSF
VAIN3WT_21	0.019	0.022	0.454	0.019	0.019	6.590	X	X	X	X	X	X	78	RBD	4	IGHV3-30	CVSFSASGTYPLYFDYW	IGKV1-33	CHQYDNLPQTF
VAIN3O_08	0.119	0.016	0.730	0.044	0.098	8.565	X	X	X	X	X	X	10	RBD	4	IGHV3-9	CAKDLHSGSYLMAHDAFDVW	IGLV1-51	CGTWDSSLSAGVS
VAIN1D_02	3.956	3.792	2.104	1.573	3.262	4.502	X	X	X	X	X	X	23	RBD	4	IGHV3-23	CAKEGPYYCSSSSCYVGIFDFW	IGKV1-13	CQQFQRYSFTF
VAIN1D_21	0.308	0.017	0.202	0.125	0.260	0.406	X	X	X	X	X	X	0	RBD	4	IGHV5-51	CARSRSSDYFDYW	IGKV1-5	CQQYKSYSKTF
VAIN2WT_12	8.648	11.377	5.392	13.725	13.283	7.194	X	X	X	X	X	X	26	RBD	4	IGHV3-30	CAKGAGPYCSAGDCLRQGLDYW	IGKV1-33	CQQYDNLPLTF
VAIN1WT_36	0.001	>50	0.005	0.339	>50	>50	X	X	X	X	X	X	83	RBD	4	IGHV3-21	CARNLGASAGVNIIHYFYHYMDVW	IGLV2-23	CCSYAGTGSPWVF
VAIN2D_36	15.179	49.955	>50	9.002	9.002	11.919	X	X	X	X	X	X	60	NTD	5	IGHV4-34	CARGLLRIFIPVGGVGGFDSW	IGKV3-15	CQQYKYWPPITF
VAIN2D_16	0.110	>50	>50	24.364	>50	10.860	X	X	X	X	X	X	54	NTD	5	IGHV4-34	CARYLQWLVPPGFDPW	IGKV1-39	CQQSYSTPRTF
VAIN1D_06	0.331	0.013	0.018	>15	0.0003	0.002	0.023	0.0004	>50	0.004	>50	>50	52	NTD	6	IGHV1-24	CATSPAVAGTMEDYSYYYGMDVW	IGKV2-24	CMQATQFPITF
VAIN3WT_06	3.043	0.157	>50	0.037	20.162	37.554	1.000	27.488	31.302	46.588	>50	>50	0	NTD	6	IGHV3-7	CARGGGHPWYYSSSGNFPPLPKADLDYW	IGLV1-44	CVAWDDSLKGSWVF
VAIN3O_10	0.772	0.004	>50	0.004	>50	>50	X	X	X	X	X	X	49	NTD	6	IGHV3-7	CTRDASNWGEWLRNSYYYYMDVW	IGLV1-44	CAAWDEGLNTWMF
VAIN1WT_13	0.033	1.138	0.045	2.185	13.421	13.950	X	X	X	X	X	X	0	NTD	x	IGHV3-7	CARDAYESSGWPVAFDIW	IGKV3-20	CQQYGSHF
VAIN1WT_46	0.014	>50	14.084	>50	>50	>50	X	X	X	X	X	X	20	NTD	x	IGHV4-39	CTRYLRGYSEDFPFDIW	IGKV1-17	CLQYNSYPHTF

Supplementary Table S4: Mutations present in variant Spikes. Unique mutations are highlighted in red. Related to Figure 3.

Variant	Spike Mutations
**Beta (B.1.351)**	L18F, D80A, D215G, (Del 241-243), K417N, E484K, N501Y, D614G, A701V
**Delta (B.1.617.2)**	T19R, G142D, 156del, 157del, R158G, L452R, T478K, D614G, P681R, D950N
**Omicron (BA.1)**	A67V, 69-70del, T95I, GVYY142-145D, NL211-212I, ins214EPE, G339D, S371L, S373P, S375F, K417N, N440K, G446S, S477N, T478K, E484A, Q493R, G496S, Q498R, N501Y, Y505H, T547K, D614G, H655Y, N679K, P681H, N764K, D796Y, N856K, Q954H, N969K, L981F
**Omicron (BA.2)**	T19I, LPPA24-27S, G142D, V213G, G339D, S371F, S373P, S375F, T376A, D405N, R408S, K417N, N440K, S477N, T478K, E484A, Q493R, Q498R, N501Y, Y505H, D614G, H655Y, N679K, P681H, N764K, D796Y, Q954H, N969K
**Omicron (BA.4)**	T19I, LPPA24-27S, Del 69-70, G142D, V213G, G339D, S371F, S373P, S375F, T376A, D405N, R408S, K417N, N440K, L452R, S477N, T478K, E484A, F486V, Q498R, N501Y, Y505H, D614G, H655Y, N679K, P681H, N764K, D796Y, Q954H, N969K
**Omicron (BA.2.75.2)**	T19I, LPPA24-27S, G142D, K147E, W152R, F157L, I210V, V213G, G257S, G339H, S371F, S373P, S375F, T376A, D405N, R408S, K417N, N440K, G446N, N460K, S477N, T478K, E484A, Q498R, N501Y, Y505H, D614G, H655Y, N679K, P681H, N764K, D796Y, Q954H, N969K
**Omicron (BQ1.1)**	T19I, LPPA24-27S, H69del, V70del, V213G, G142D, G339D, R346T, S371F, S373P, S375F, T376A, D405N, R408S, K417N, N440K, K444T, L452R, N460K, S477N, T478K, E484A, F486V, Q498R, N501Y, Y505H, D614G, H655Y, N679K, P681H, N764K, D796Y, Q954H, N969K
**Omicron (XBB)**	T19I, LPPA24-27S, V83A, G142D, Del144, H146Q, Q183E, V213E, G252V, G339H, R346T, L368I, S371F, S373P, S375F, T376A, D405N, R408S, K417N, N440K, V445P, G446S, N460K, S477N, T478K, E484A, F486S (**XBB1.5:** F486P**),** F490S, Q498R, N501Y, Y505H, D614G, H655Y, N679K, P681H, N764K, D796Y, Q954H, N969K

References:

1 Scheid, J. F. *et al.* Broad diversity of neutralizing antibodies isolated from memory B cells in HIV-infected individuals. *Nature* **458**, 636-640, doi:10.1038/nature07930 (2009).

2 Tiller, T. *et al.* Efficient generation of monoclonal antibodies from single human B cells by single cell RT-PCR and expression vector cloning. *J Immunol Methods* **329**, 112-124, doi:10.1016/j.jim.2007.09.017 (2008).

3 von Boehmer, L. *et al.* Sequencing and cloning of antigen-specific antibodies from mouse memory B cells. *Nat Protoc* **11**, 1908-1923, doi:10.1038/nprot.2016.102 (2016).

4 Briney, B., Inderbitzin, A., Joyce, C. & Burton, D. R. Commonality despite exceptional diversity in the baseline human antibody repertoire. *Nature* **566**, 393-397, doi:10.1038/s41586-019-0879-y (2019).
